# Supplementary material for: Lowering of the singlet-triplet energy gap via intramolecular exciton-exciton coupling
Source: Nat Commun. 2024 Oct 8;15:8705. doi: 10.1038/s41467-024-53122-7 (PMC11461719; doi:10.1038/s41467-024-53122-7)
Supplement: Supplementary file 1 — Supplementary Information [file 41467_2024_53122_MOESM1_ESM.pdf]

Supplementary Information for

Lowering of the singlet-triplet energy gap *via* intramolecular exciton-exciton coupling.

Clara Schäfer[a], Rasmus Ringström[b], Jörg Hanrieder[c,d], Martin Rahm[b], Bo Albinsson[b], Karl Börjesson[a,*]

[a] Department of Chemistry and Molecular Biology, University of Gothenburg, Box 462, 405 30 Gothenburg, Sweden

[b] Department of Chemistry and Chemical Engineering, Chalmers University of Technology, Kemivägen 10, 412 96 Gothenburg, Sweden.

[c] Department of Psychiatry and Neurochemistry, Institute of Neuroscience and Physiology, Sahlgrenska Academy at the University of Gothenburg, Mölndal Hospital, House V3, 43180 Mölndal, Sweden.

[d] Department of Neurodegenerative Disease, Queen Square Institute of Neurology, University College London, London WC1N 3BG, United Kingdom.

Email: karl.borjesson@gu.se

Table of Contents

Supplementary Note 1: Synthetic procedures 2

Monomer 2

Dimer, trimer, and tetramer 2

Supplementary Note 2: Diffusion experiment 5

Supplementary Note 3: Calculating the transition dipole moment from absorption spectra 6

Supplementary Note 4: Analysis of exciton coupling using a point dipole approximation 7

Supplementary Note 5: Modelling of temperature resolved emission of the Monomer 8

Supplementary Note 6: Electrochemical analysis 10

Supplementary Note 7: Simulating the yield of fluorescence and intersystem crossing. 11

Supplementary Note 8: Analysing the CS recombination using fs-transient absorption and Marcus theory. 13

Supplementary Figures 15

Chemical characterization 15

Molar absorptivities 25

Geometry optimized structures 26

Steady state absorption and emission spectra in various solvents 27

Time resolved emission 32

Temperature dependent emission 37

Electrochemistry 38

Nanosecond transient absorption 39

Femtosecond transient absorption 43

Analyzing recombination using Marcus theory 45

Supplementary Tables 46

Supplementary References 48

Supplementary Note 1: Synthetic procedures

Monomer

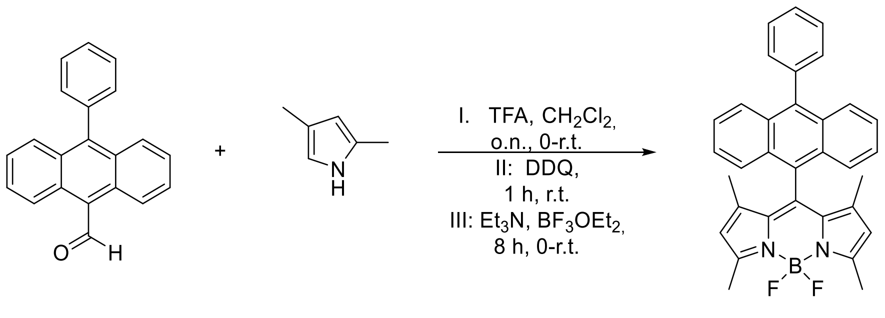

3

The phenyl-anthracene-BODIPY dyad was synthesized following the literature procedure.[1]

10-phenylanthracene-9-carbaldehyde (1.38 g, 4.9 mmol) and 2, 4-dimethylpyrrole (1.2 mL, 11.3 mmol) were added to 150 ml anhydrous CH_2Cl_2, the flask was charged with Ar. Three drops of trifluoroacetic acid were added under ice-cold condition. Then, the mixture was stirred at room temperature overnight. Then DDQ (1.2 g) was added and remained for 20 minutes. Subsequently, Et_3N (10 mL) and $BF_3{\cdot}Et_2O$ (10 mL) were added under ice-cold condition, and the reaction mixture was stirred for an additional 8 h. The mixture was poured into 200 mL water, and the organic layer was collected and evaporated under reduced pressure. The crude product was purified by silica gel column chromatography (CH_2Cl_2:PE = 10:6). The red band in the column was collected, and the solvent was removed under reduced pressure to give a red solid. Yield: 526.8 mg, 21.5%. Measured in $CDCl_3$: ^{1}H NMR (800 MHz, $CDCl_3$) δ = 7.96 (d, *J* = 8.7 Hz, 2H), 7.68 (d, *J* = 8.8 Hz, 2H), 7.62 (t, *J* = 7.3 Hz, 2H), 7.57 (t, *J* = 7.5 Hz, 1H), 7.47 (d, *J* = 6.6 Hz, 2H), 7.43 – 7.39 (m, 2H), 7.38 – 7.33 (m, 2H), 5.93 (s, 2H), 2.64 (s, 6H, $-CH_3$), 0.75 (s, 6H, $-CH_3$). Recorded data are in accordance with the literature.[1] Measured data in CD_2Cl_2: ^{1}H NMR (700 MHz, CD_2Cl_2) δ = 7.96 (dt, *J* = 8.8, 1.0 Hz, 2H), 7.73 (dt, *J* = 8.7, 1.0 Hz, 2H), 7.70 – 7.65 (m, 2H), 7.65 – 7.60 (m, 1H), 7.53 – 7.49 (m, 2H), 7.46 (ddd, *J* = 8.7, 6.4, 1.3 Hz, 2H), 7.41 (ddd, *J* = 8.8, 6.4, 1.3 Hz, 2H), 6.00 (s, 2H), 2.64 (s, 6H, $-CH_3$), 0.79 (s, 6H, $-CH_3$).z ^{13}C NMR (176 MHz, CD_2Cl_2) δ = 155.8, 143.0, 139.6, 139.4, 138.3, 132.4, 131.3, 130.0, 129.3, 128.4, 128.1, 127.8, 127.1, 126.7, 125.6, 125.0, 121.1, 53.7, 53.6, 53.4, 53.3, 53.1, 14.4, 13.1. ^{19}F NMR (659 MHz, CD_2Cl_2) δ = -145.99, -146.04, -146.09, -146.14.

Dimer, trimer, and tetramer

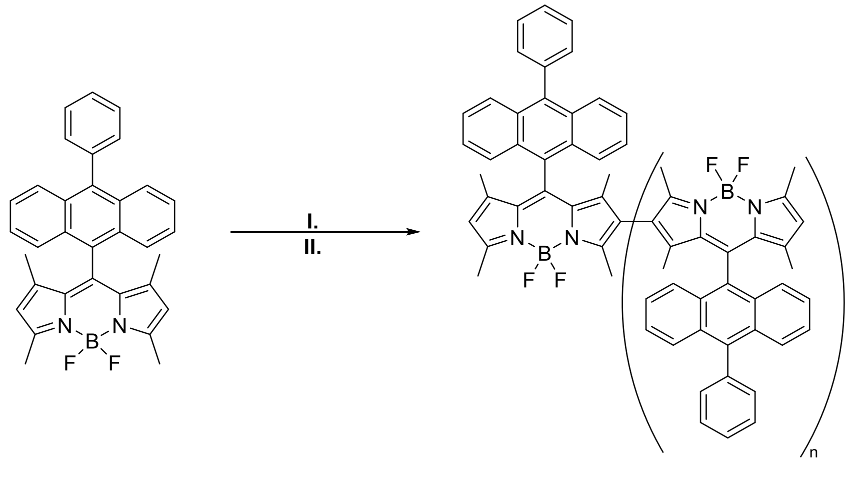

I. PIFA, $BF_3{\cdot}OEt_2$, DCM, -78°C - r.t, 1 h. II.) $FeCl_3$ anhydrous, DCM anhydrous, r.t., 25 min.

Oligomerization using PIFA:[2] To a stirred solution of phenyl-anthracene-BODIPY dyad (100 mg, 0.200 mmol) in CH_2Cl_2 (10 mL), PIFA (49.2 mg, 0.115 mmol) and BF_3OEt_2 (0.030 mL) were quickly added at -78°C. The reaction mixture was then stirred for 30 min and the reaction progress was checked by TLC, but only very little conversion was observed. The reaction was then stirred at room temperature for 1 hour. Saturated aqueous sodium hydrogen carbonate was added to the mixture. The aqueous phase was extracted with CH_2Cl_2. The organic phase was then dried over anhydrous $MgSO_4$ and evaporated to dryness. The dimer was isolated as a wine-red solid (8mg, 0.008 mmol, 8 %) after two consecutive purification steps. The first step was a purification using column chromatography on silica with hexane/DCM (80-100 %). The second purification was a second SiO_2 column using hexane/DCM (80 %).

Alternatively:[3] To a solution of phenyl-anthracene-BODIPY dyad (130 mg, 0.260 mmol) in Dry CH_2Cl_2 (40 mL) anhydrous $FeCl_3$ (148 mg, 0.910 mmol, 3.5 equiv.) was added at room temperature. The orange solution promptly turns dark purple. After stirring for 20 min the reaction was quenched by the addition of MeOH (75 mL). The organic phase was first washed with H_2O (3 x 100 mL) and subsequently dried over Na_2SO_4. The solvent was removed under reduced pressure. The monomer (30 mg, 0.060 mmol, 23 %), dimer (22 mg, 0.022 mmol, 17 %), trimer 1 (2.5 mg, 0.0017 mmol, 2 %) and trimer 2 (1.0 mg, 0.00067 mmol, <1 %) and the tetramer (1 mg, 0.00050 mmol, < 1 %) were isolated, following the below described purification steps.

The crude product was purified using column chromatography on silica with hexane/DCM (80-100 %). The residual phenyl-anthracene-BODIPY dyad was eluted first as a yellow, green-fluorescent fraction, followed by the dimer, a pink, orange-fluorescent fraction. A third purple, red-fluorescent fraction was eluted next using DCM with 1 % MeOH. The fractions were further purified using a second SiO_2 column using hexane/DCM (80 %) in case of the dimer. The third, purple fraction was further purified with size exclusion column chromatography (SEC) using Bio-Beads S-X3 Support as a stationary phase and DCM as eluent, the single fractions from SEC were further purified using preparative TLC. This yielded two different atropisomers of the trimer and one isomer the tetramer.

The **dimer** was isolated as a wine-red solid (8mg, 0.008 mmol, 8 % using PIFA) (22 mg, 0,022 mmol, 17 %, using anhydrous $FeCl_3$). Measured in $CDCl_3$: ^{1}H NMR (700 MHz, $CDCl_3$) δ = 8.00 (d, *J* = 8.6 Hz, 2H), 7.89 – 7.83 (m, 2H), 7.69 (d, *J* = 8.8 Hz, 2H), 7.68 – 7.54 (m, 8H), 7.49 – 7.41 (m, 6H), 7.39 (ddd, *J* = 8.8, 6.4, 1.2 Hz, 2H), 7.32 – 7.27 (m, 4H), 5.93 (s, 2H), 2.64 (s, 6H, $-CH_3$), 2.39 (s, 6H, $-CH_3$), 0.73 (s, 6H, $-CH_3$), 0.47 (s, 6H, $-CH_3$). ^{13}C NMR (176 MHz, $CDCl_3$) δ = 156.2, 155.0, 143.3, 140.9, 139.4, 139.3, 138.3, 132.8, 132.2, 131.3, 131.3, 129.9, 129.9, 129.4, 129.2, 128.5, 128.4, 128.2, 127.8, 127.1, 127.1, 126.7, 126.5, 125.7, 125.5, 125.1, 125.0, 124.6, 121.4, 77.2, 77.0, 76.8, 29.7, 26.9, 14.8, 13.5, 11.9. Measured in CD_2Cl_2: ^{1}H NMR (700 MHz, CD_2Cl_2) δ 8.00 (dt, J = 8.6, 1.0 Hz, 2H), 7.88 – 7.83 (m, 2H), 7.71 (dt, J = 8.8, 1.0 Hz, 2H), 7.68 – 7.62 (m, 6H), 7.63 – 7.58 (m, 2H), 7.51 – 7.45 (m, 6H), 7.41 (ddd, J = 8.8, 6.4, 1.2 Hz, 2H), 7.34 – 7.29 (m, 4H), 5.98 (s, 2H), 2.61 (s, 6H, $-CH_3$), 2.37 (s, 6H, $-CH_3$), 0.73 (s, 6H, $-CH_3$), 0.50 (s, 6H, $-CH_3$). ^{13}C NMR (176 MHz, CD_2Cl_2) δ 156.21, 154.95, 143.30, 140.93, 139.56, 139.37, 138.26, 132.68, 132.27, 131.27, 129.99, 129.91, 129.35, 129.22, 128.40, 128.12, 127.74, 127.06, 126.72, 126.54, 125.60, 125.44, 124.93, 124.79, 124.54, 121.31, 29.68, 14.46, 13.87, 13.14, 11.62. ^{19}F NMR (659 MHz, CD_2Cl_2) δ = -146.20, -146.24, -146.29, -146.34, -146.39. HRMS: (ESI+) m/z calcd. For $(M+H)^+$ $C_{66}H_{53}B_2F_4N_4$: 997.4465; found: 997.4421. MALDI: (Reflector positive mode, mass range 400-3200 Da, MS Dried Droplet laser mode, 200 laser shots and 59% laser power) Main peak: m/z 998.408. M.p.: no melting observed only decomposition > 250 °C. IR ν_{max}/cm^{-1}: 2960, 2922, 2857, 2363, 2332, 1535, 1462, 1441, 1400, 1362, 1306, 1184, 1163, 1119, 1079, 978, 778, 704, 647, 613, 600, 579, 493.

Two atropisomers of the **trimer** were isolated as purple solids, trimer 1 (2.5 mg, 0.0017 mmol, 2 %) and trimer 2 (1.0 mg, 0.00067 mmol, <1 %). **trimer 1:** ^{1}H NMR (700 MHz, CD_2Cl_2) δ = 7.98 (dt, J = 8.7,

1.0 Hz, 2H), 7.93 – 7.88 (m, 2H), 7.86 – 7.82 (m, 2H), 7.71 (dt, J = 8.8, 1.1 Hz, 2H), 7.66 – 7.59 (m, 13H), 7.48 – 7.46 (m, 6H), 7.44 – 7.40 (m, 4H), 7.35 – 7.29 (m, 8H), 5.97 (s, 2H), 2.60 (s, 6H, -CH_3), 2.34 (s, 6H, -CH_3), 2.32 (s, 6H, -CH_3), 0.73 (s, 6H, -CH_3), 0.49 (s, 6H, -CH_3), 0.45 (s, 6H, -CH_3). ^{13}C NMR (151 MHz, CD_2Cl_2) δ = 15z6.3, 155.4, 154.8, 143.4, 141.2, 140.9, 139.6, 139.4, 138.3, 138.2, 132.7, 132.5, 132.2, 131.3, 131.3, 130.0, 129.9, 129.3, 129.2, 129.2, 128.4, 128.4, 128.4, 128.1, 128.1, 127.7, 127.1, 127.0, 126.7, 126.6, 126.5, 125.6, 125.5, 125.4, 124.9, 124.8, 124.4, 121.3, 14.5, 13.9, 13.2, 13.1, 11.6, 11.6. ^{19}F NMR (564 MHz, CD_2Cl_2) δ = -146.20, -146.26, -146.31, -146.37, -146.43, -146.48, -146.53, -146.59, -146.65. MALDI: (Reflector positive mode, mass range 400-3200 Da, MS Dried Droplet laser mode, 200 laser shots and 59% laser power) Main peak: m/z 1496.596. M.p.: no melting observed only decomposition > 250 °C. IR $ν_{max}$/cm^{-1}: 2959, 2918, 2850, 2362, 2338, 1540, 1457, 1396, 1305, 1163, 1116, 987, 778, 668, 649, 478. **trimer 2:** ^{1}H NMR (700 MHz, CD_2Cl_2) δ = 8.05 (dt, J = 8.6, 1.0 Hz, 1H), 7.98 (dt, J = 8.7, 1.0 Hz, 2H), 7.86 – 7.81 (m, 2H), 7.76 (dt, J = 8.6, 1.0 Hz, 1H), 7.69 (dt, J = 8.7, 1.0 Hz, 2H), 7.66 – 7.57 (m, 13H), 7.49 – 7.37 (m, 14H), 7.29 (dq, J = 6.3, 3.2 Hz, 4H), 5.97 (s, 2H), 2.60 (s, 6H, -CH_3), 2.36 (s, 6H, -CH_3), 2.35 (s, 6H, -CH_3), 0.72 (s, 6H, -CH_3), 0.46 (s, 6H, -CH_3), 0.45 (s, 6H, -CH_3). ^{13}C NMR (151 MHz, CD_2Cl_2) δ = 156.3, 155.4, 154.9, 143.3, 141.2, 140.8, 139.5, 139.4, 138.2, 132.2, 131.3, 130.0, 129.9, 129.3, 129.2, 128.4, 128.2, 128.1, 127.7, 127.6, 127.0, 127.0, 126.8, 126.7, 126.5, 126.4, 126.1, 125.6, 125.6, 125.4, 124.9, 124.8, 124.6, 121.3, 53.8, 53.6, 53.4, 53.3, 53.1, 29.7, 22.7, 14.5, 13.9, 13.1, 11.6, 11.6. ^{19}F NMR (564 MHz, CD_2Cl_2) δ = -146.20, -146.25, -146.31, -146.36, -146.41, -146.42, -146.46, -146.56, -146.66, -146.72. MALDI: (Reflector positive mode, mass range 400-3200 Da, MS Dried Droplet laser mode, 200 laser shots and 59% laser power) Main peak: m/z 1496.600. M.p.: no melting observed only decomposition > 250 °C. IR $ν_{max}$/cm^{-1}: 2958, 2918, 2851, 2362, 2338, 1540, 1457, 1378, 1305, 1163, 1116, 982, 668.

One isomer of the **tetramer** was isolated as a purple solid (1 mg, 0.00050 mmol, < 1 %) ^{1}H NMR (700 MHz, CD_2Cl_2) δ 7.98 (dt, *J* = 8.6, 1.1 Hz, 2H), 7.92 – 7.87 (m, 4H), 7.85 – 7.81 (m, 2H), 7.70 (dt, *J* = 8.8, 0.9 Hz, 2H), 7.66 – 7.59 (m, 18H), 7.48 – 7.45 (m, 6H), 7.44 – 7.39 (m, 6H), 7.33 – 7.29 (m, 12zH), 5.97 (s, 2H), 2.59 (s, 6H, -CH_3), 2.33 (s, 6H, -CH_3), 2.32 (s, 6H, -CH_3), 2.29 (s, 6H, -CH_3), 0.72 (s, 6H, -CH_3), 0.48 (s, 6H, -CH_3), 0.44 (s, 6H, -CH_3), 0.43 (s, 6H, -CH_3). ^{13}C NMR (151 MHz, CD_2Cl_2) δ = 155.5, 154.7, 143.4, 141.2, 141.1, 139.6, 139.4, 138.2, 132.7, 132.5, 132.5, 131.2, 130.0, 129.9, 129.3, 129.2, 128.4, 128.1, 127.7, 127.0, 126.7, 126.6, 126.5, 125.6, 125.5, 124.9, 124.8, 22.7, 14.5, 13.9, 13.1, 11.6. (Due to the low concentration of the sample not all ^{13}C-peaks could be resolved.) ^{19}F NMR (564 MHz, CD_2Cl_2) δ = -146.21, -146.21, -146.26, -146.32, -146.32, -146.38, -146.43, -146.50, -146.55, -146.62, -146.67. MALDI: (Reflector positive mode, mass range 400-3200 Da, MS Dried Droplet laser mode, 200 laser shots and 59% laser power) Main peak: m/z 1994.771. M.p.: no melting observed only decomposition > 250 °C. IR $ν_{max}$/cm^{-1}: 2959, 2922, 2853, 2362, 2338, 1540, 1522, 1473, 1457, 1419, 1308, 1163, 1118, 991, 778, 668, 649, 478.

In the ^{1}H-NMR spectra, the CH_3 peaks are indicative for the size of the oligomer. The monomer has 2 unique CH_3-peaks, the dimer four, the trimer six, and the tetramer 8. The two atropisomers of the trimer show different patterns in separation of those peaks. The isolated tetramer shows only one kind of peak pattern, indicating that only one isomer was isolated.

Supplementary Note 2: Diffusion experiment

The diffusion NMR experiments were conducted to estimate the physical size of the oligomers. The diffusion experiment was a pseudo 2D experiment with 16 gradient strength The pseudo–2D diffusion spectra were recorded using the default Bruker pulse sequence *ledbpgp2s*[4] with 16 experiments and linearly changing gradient strength. The details of the diffusion experiments are shown in Supplementary Table 1. The experiments were all recorded at 298K and the gradient calibration constant (GCC) was determined using the known diffusion coefficient ($1.91 \cdot 10^{-9}$ m^2/s) of the standard Bruker doped water sample (Z10906). The diffusion coefficients were extracted for several peak integrals which then were fitted using a python program. The error of the diffusion coefficient was then determined from these individual values as an average for a certain oligomer together with its error.

The results of the data analysis are shown in Supplementary Table 2. The reported experimental diffusion coefficients are the averages over all of the integrals taken in the spectra and the error is the standard deviation of these values. This method however probably underestimates the error in the measured value. The measured diffusion coefficients agree very well with the model of the samples namely the monomer contains the monomer, the dimer the dimer and so on. There are two types of trimers labelled trimer–1 and trimer–2. Their experimental diffusion coefficient differs slightly but this could be due to measurement error.

In the HYDRO++10 simulations the molecules are represented by a bead model which in this case are beads placed on the heavy atoms so that they do not overlap with each other (with a radius of 0.7˚Å). In a HYDROSUB calculation the model is built differently and in this case the beads can overlap. The model is built from predefined prolates and oblates. In this case the monomer was built from 2 prolates with a long semi axis of 6.8 and 7.4˚Å and a short semi axis of 1.2 Å and from an oblate with semi long axis of 2.0˚Å and semi short axis of 1.0 Å. These were then placed such that the prolates were perpendicular to each other and the oblate was perpendicular to one of the prolate representing the single benzene ring. The size of the axes was taken from the XYZ representation of the molecule in the Avogadro software and then they were slightly adjusted to match the diffusion coefficient of the monomer. After this the same moiety was repeated twice for the dimer, three times for the trimer and four times for the tetrameter.

Supplementary Note 3: Calculating the transition dipole moment from absorption spectra

The transition dipole moment of the low energy transitions of the monomer, dimer, trimer and tetramer were determined from the molar absorptivity spectra using the following formula:[5]

$$\mu = 9.58 \cdot 10^{-2} \left(\frac{(2n^2+1)^2}{9n^3} \int \frac{\varepsilon(v)}{v} dv \right)^{1/2} \qquad \text{(S1)}$$

Where n is the refractive index (DCM = 1.4), ν represents wavenumber (in cm^{-1}), and μ is the transition dipole moment in Debye. First the integrand over the low energy transition in Equation S1 was evaluated to 5000 M^{-1} cm^{-1}, 11500 M^{-1} cm^{-1}, 13900 M^{-1} cm^{-1}, and 17300 M^{-1} cm^{-1}, for the monomer to tetramer, respectively (Supplementary Figure 20). After this, the transition dipole moments were calculated using Equation S1 to 6.71 D, 10.2 D, 11.2, and 12.5 D, respectively.

Supplementary Note 4: Analysis of exciton coupling using a point dipole approximation

The interaction energy between the S_0-S_1 transitions of two BODIPY moieties was determined using a point dipole approximation,[6] using the notation from Yoon et al.[7]

$$H_{ij} = \frac{5.04 f^2 |\mu_i||\mu_j| \kappa}{\varepsilon_r |R_{ij}|^3} \tag{S2}$$

$$\kappa = \cos\Theta - 3\cos\alpha\cos\beta \tag{S3}$$

$$f = \frac{\varepsilon_r + 2}{3} \tag{S4}$$

Where H_{21} is the interaction energy between moiety i and j (the number index corresponds to the BODIPY position in the oligomer), and *f* is the Lorentz correction factor. The transition dipole moment (μ) of the S_0-S_1 transition is 6.7 Debye (Supplementary Notes 3). The angles $\theta_{ij}=\alpha_{ij}=\beta_{ij}=0$, which gives an orientation factor, κ, of -2. The relative dielectric constant of DCM is 9.1. The center-center distances between BODIPY moieties were taken from the geometry optimized structures and are R_{12}=0.8202 nm, R_{12}=1.6397 nm and R_{12}=2.4598 nm. Using these values, the interaction energies between the different BODIPY moieties in the oligomer were calculated to $H_{21}=H_{12}$=-1236 cm^{-1}, $H_{31}=H_{13}$=-154.7 cm^{-1}, and $H_{41}=H_{41}$=-45.83 cm^{-1}.

The energies of the states formed by exciton coupling can be calculated by solving the following secular determinants.

$$\begin{vmatrix} H_{11}-E_B & H_{21} \\ H_{12} & H_{22}-E_B \end{vmatrix} = 0 \qquad \text{dimer} \tag{S5}$$

$$\begin{vmatrix} H_{11}-E_B & H_{21} & H_{31} \\ H_{12} & H_{22}-E_B & H_{32} \\ H_{13} & H_{23} & H_{33}-E_B \end{vmatrix} = 0 \qquad \text{trimer} \tag{S6}$$

$$\begin{vmatrix} H_{11}-E_B & H_{21} & H_{31} & H_{41} \\ H_{12} & H_{22}-E_B & H_{32} & H_{42} \\ H_{13} & H_{23} & H_{33}-E_B & H_{43} \\ H_{14} & H_{24} & H_{34} & H_{44}-E_B \end{vmatrix} = 0 \qquad \text{tetramer} \tag{S7}$$

Where $H_{11}=H_{22}=H_{33}=H_{44}$ is the energy of the unperturbed $S_0 \rightarrow S_1$ transition of BODIPY (2.45 eV, 19800 cm^{-1}), and E_B are the energies of the formed hybrid states. The hybrid states of lowest energy for the dimer, trimer, and tetramer have energies of 2.297 eV (18500 cm^{-1}), 2.224 eV (17900 cm^{-1}), and 2.183 eV (17600 cm^{-1}), respectively, and are displayed in Figure 2b.

Supplementary Note 5: Modelling of temperature resolved emission of the Monomer

The temperature resolved emission was conducted in DCM and was modelled in the following way. It was assumed that the radiative as well as non-radiative rate constants from the S_1 state to the ground state (k_{S1toS0_nr} and k_{S1toS0_rad}) were the same in toluene as in DCM. This allowed these rate constants to be set from the measurements performed in toluene. The total rate from the CS state to the ground and T_1 states (k_{CStoS0}) was set to the inverse of the CS lifetime at each temperature. The S_1 and CS states were assumed to be in microscopic reversibility. For such an ideal case the ratio of the rates between the two states equals to the Boltzmann factor. When exciting the S_1 state with a constant flux (I), the rate equations describing the concentration change with time from these two states can be written as:

$$\frac{dS_1}{dt} = I - k_{S1\to S0_{nr}} \cdot S_1 - k_{S1\to S0_{rad}} \cdot S_1 - k_{S1\to CS} \cdot S_1 + k_{CS\to S1} e^{-\frac{Ea}{RT}} \cdot CS \qquad \text{(S8)}$$

$$\frac{dCS}{dt} = k_{S1\to CS} \cdot S_1 - k_{CS\to S0}(T) \cdot \mathrm{CS} - k_{CS\to S1} e^{-\frac{Ea}{RT}} \cdot \mathrm{CS} \qquad \text{(S9)}$$

Where k_{CStoS1}=k_{S1toCS} (they are equal because the two states are connected by a microscopic reversibility). At steady state conditions, this time derivative in Supplementary Equations 8&9 can be set to zero. Furthermore, the definition of the quantum yield is:

$$\Phi_F = \frac{k_{S1\to S0_{rad}} \cdot S_1}{I} \qquad \text{(S10)}$$

Thus, inserting Supplementary Equations 8&9 into 10 allows an analytical expression for the fluorescence quantum yield at steady state conditions to be obtained:

$$\Phi_F = \frac{k_{S1\to S0_{rad}} \cdot \left(k_{CS\to S0}(T) + k_{CS\to S1} e^{-\frac{Ea}{RT}}\right)}{\left(k_{S1\to S0_{nr}} + k_{S1\to S0_{rad}} + k_{S1\to CS}\right)\left(k_{CS\to S0}(T) + k_{CS\to S1} e^{-\frac{Ea}{RT}}\right) - k_{S1\to S0_{rad}} \cdot k_{CS\to S1} e^{-\frac{Ea}{RT}}} \qquad \text{(S11)}$$

The experimental fluorescence quantum yield was extracted by first deconvoluting the emission in DCM using three Gaussian functions into a fluorescence part (2 Gaussians) and a CS emission part (1 Guassian) at each measured temperature, then scaled with the absorbance, and the total emission quantum yield (5%, Table 1) at room temperature. The deconvolution also allowed to quantify an observed shift in the emission maximum of the CS emission (such a shift can be explained by changes in the refractive index of the solvent when decreasing the temperature), which was taken into account in the analysis. Furthermore, we noted that the CS emission is significantly reduced with decreasing temperature. The radiative rate constant of CS emission from TICT molecules is known to be temperature dependent, which explains such phenomena and prevents a detailed analysis of the CS emission with decreasing temperature.[8]

The values of Ea and k_{CStoS1}=k_{S1toCS} were used as fitting parameters in a process where the sum of squared residuals between the measured and calculated temperature dependent fluorescence quantum yield was minimized. The received values of Ea and k_{CStoS1}=k_{S1toCS} were 12 kJ/mol and $3.3\cdot10^{10}$ s^{-1}, respectively. These two values are matching expectations reasonably well based on the energy of the emission spectra and a decay that is not completely temporally resolved (about a factor 2 lower than our expectations) by our equipment. Regarding fitting sensitivity, Ea was found to be sensitive to the curvature of the temperature dependence, while k_{CStoS1} was found to be sensitive to Φ_F at low temperatures.

Supplementary Figure 34 show the temperature resolved absorption, emission decays, and emission, and Supplementary Figure 35 show simulated and measured fluorescence quantum yields and decays.

The theoretical quantum yield of emission shows a decrease with temperature, which is in line with the measured values. The emission decays show two clear lifetimes, one very short and one on the nanosecond timescale. It further shows a decrease of the longlived component with decreasing temperature in a similar fashion as in the experimental data (Supplementary Figure 34).

In summary, a model including a dynamic equilibrium between the S_1 and CS states do well explain some of the peculiar photophysical features of the Monomer, such as a decreasing fluorescence quantum yield with temperature, and a biexponential lifetime, with a long component that are much longer compared what is expected based on the quantum yield of emission and the radiative rate constant of the S_1 state.

Supplementary Note 6: Electrochemical analysis

To investigate whether the energetics of the CS state is influenced by oligomerization, the redox potentials of the monomer and the dimer were determined in DCM. Limited solubility in ACN prevented experimental determination of the CS state energy, and we therefore relied on the Rehm-Weller equation for this value. The monomer shows a reversible one-electron oxidation and reduction at + 0.71 V and – 1.73 V vs. Fc/Fc^+, respectively in DCM (Supplementary Figure 42a, Table S4). They correlate very well with the previously reported redox potentials of the monomer,[9] where the reduction was assigned to the BODIPY and oxidation to the anthracene moieties. The dimer in DCM shows two reversible one-electron waves for reduction (Supplementary Figure 42b, Table S4). However, the first reduction signal at - 1.71 V is almost identical with the one observed for the monomer. This indicates that the reduction of the monomeric unit of the dimer, is almost identical to the monomer. The reduction potential of the second unit is influenced by the first reduction. This trend was observed previously for the redox potentials of non-conjugated BODIPY dimers and trimers linked in the β-position.[2] It indicates that the BODIPY moieties do have some electronic communication. However, a large change such as a reduction, resulting in a strong electron donating effect, is needed to significantly perturb the energetics of the neighboring unit. In contrast to the reduction, only one oxidation potential for the dimer was observed in close proximity to the end of the solvent window. This can be due to the limitation of solvent window or because the anthracene units are too far away from each other to communicate electronically. A small shift of 80 meV for the oxidation potential was seen. It is unlikely that this is caused by anthracene-anthracene interactions, because of their large distance to each other. However, the oxidation peak is not well resolved, and the error margins are therefore large.

The energies of the charge separated states (E_{CS}) can be calculated with Supplementary Equation 12, where the static Coulombic energy (ΔG_S) can be calculated using Supplementary Equation 13.

$$E_{CS} = e[E_{ox} - E_{red}] + \Delta G_S \tag{S12}$$

$$\Delta G_S = -\frac{e^2}{4\pi\varepsilon_S\varepsilon_0 R_{CC}} - \frac{e^2}{8\pi\varepsilon_0}\left(\frac{1}{R_D} + \frac{1}{R_A}\right)\left(\frac{1}{\varepsilon_{ref}} - \frac{1}{\varepsilon_S}\right) \tag{S13}$$

Where, e = electronic charge, E_{ox} = half-wave potential for one-electron oxidation of the electron-donor unit (anthracene$^{0/\bullet+}$) , E_{red} = halfwave potential for one-electron reduction of the electron-acceptor moiety (BODIPY $^{0/\bullet-}$); ε_S = static dielectric constant of the solvent, R_{CC} = center-to-center separation distance between the electron donor (anthracene) and electron acceptor (BODIPY), determined by the DFT optimization of the geometry, R_D is the radius of the electron donor, R_A is the radius of the electron acceptor, ε_{ref} is the static dielectric constant of the solvent used for the electrochemical studies, ε_0 is permittivity of free space.[9]

The solvents used in the calculation of free energy of the electron transfer is, toluene (ε_S = 2.38), DCM (ε_S = 8.93) and ACN (ε_S = 37.5).

Supplementary Note 7: Simulating the yield of fluorescence and intersystem crossing.

The yields of emission and intersystem crossing were simulated from known measurables in order to gain a thorough understanding of the observed photophysics in the series. A similar rate equation model as the one used for modelling the temperature dependent emission was constructed. It is based on a three-level system, the S_1, CS, and T_1 states, of which the S_1 and CS states are in dynamic equilibrium with each other (Fig. 5a). The S_1 state was excited and the analytical expressions for the yields of fluorescence and intersystem crossing was constructed. Figure 5a can be summarized with the following two rate equations.

$$\frac{dS_1}{dt} = I - k_{S1\to S0_{nr}} \cdot S_1 - k_{S1\to S0_{rad}} \cdot S_1 - k_{S1\to CS}(Ea,T) \cdot S_1 + k_{CS\to S1}(Ea,T) \cdot CS \qquad \text{(S14)}$$

$$\frac{dCS}{dt} = k_{S1\to CS}(Ea,T) \cdot S_1 - k_{CS\to S0} \cdot \mathrm{CS} - k_{CS\to T1} \cdot \mathrm{CS} - k_{CS\to S1}(Ea,T) \cdot \mathrm{CS} \qquad \text{(S15)}$$

The steady state approximation can be used to set the derivatives to zero:

$$0 = I - k_{S1\to S0_{nr}} \cdot S_1 - k_{S1\to S0_{rad}} \cdot S_1 - k_{S1\to CS}(Ea,T) \cdot S_1 + k_{CS\to S1}(Ea,T) \cdot CS \qquad \text{(S16)}$$

$$0 = k_{S1\to CS}(Ea,T) \cdot S_1 - k_{CS\to S0} \cdot \mathrm{CS} - k_{CS\to T1} \cdot \mathrm{CS} - k_{CS\to S1}(Ea,T) \cdot \mathrm{CS} \qquad \text{(S17)}$$

and the definitions of the fluorescence quantum and intersystem crossing yields are:

$$\Phi_F = \frac{k_{S1\to S0_{rad}} \cdot S_1}{I} \qquad \text{(S18)}$$

$$\Phi_{ISC} = \frac{k_{CS\to T1} \cdot \mathrm{CS}}{I} \qquad \text{(S19)}$$

where now S_1 and CS denotes the concentrations of these states at steady state. Inserting S16 into S19 gives the quantum yield of intersystem crossing as a function of the S_1 and CS concentrations.

$$\Phi_{ISC} = \frac{k_{CS\to T1} \cdot \mathrm{CS}}{k_{S1\to S0_{nr}} \cdot S_1 + k_{S1\to S0_{rad}} \cdot S_1 + k_{S1\to CS} \cdot S_1 - k_{CS\to S1} e^{-\frac{Ea}{RT}} \cdot CS} \qquad \text{(S20)}$$

From equation S17 we can formulate an expression of the S_1 concentration as a function of the CS concentration:

$$S_1 = \frac{1}{k_{S1\to CS}} \left(k_{CS\to S0}(T) + k_{CS\to T1} + k_{CS\to S1} e^{-\frac{Ea}{RT}} \right) \cdot \mathrm{CS} \qquad \text{(S21)}$$

Now, inserting S21 into S20 gives an expression for the quantum yield of intersystem crossing without involving the S_1 concentration.

$$\Phi_{ISC} = \frac{k_{CS\to T1} \cdot \mathrm{CS}}{\left(k_{S1\to S0_{nr}} + k_{S1\to S0_{rad}} + k_{S1\to CS}\right) \cdot \frac{1}{k_{S1\to CS}} \left(k_{CS\to S0}(T) + k_{CS\to T1} + k_{CS\to S1} e^{-\frac{Ea}{RT}} \right) \cdot \mathrm{CS} - k_{CS\to S1} e^{-\frac{Ea}{RT}} \cdot CS} \qquad \text{(S22)}$$

By simplifying S22, one gets:

$$\Phi_{ISC} = \frac{k_{CS\to T1} \cdot k_{S1\to CS}}{\left(k_{S1\to S0_{nr}} + k_{S1\to S0_{rad}} + k_{S1\to CS}\right) \cdot \left(k_{CS\to S0}(T) + k_{CS\to T1} + k_{CS\to S1} e^{-\frac{Ea}{RT}} \right) - k_{S1\to CS} \cdot k_{CS\to S1} e^{-\frac{Ea}{RT}}} \qquad \text{(S23)}$$

Several of the rate constants can be directly calculated from experimental data, and others were approximated. The rate constants of k_{S1toS0_nr} and k_{S1toS0_rad} were approximated from the fluorescence quantum yield and lifetime in toluene solution. The CS state is too high in energy to be populated from S_1 in toluene solution, and the assumption done in this approximation is that k_{S1toS0_nr} is solvent

independent. The S_1 and CS states are assumed to be in microscopic reversibility, and that the rate difference between these states equals to an energetic penalty that equals the energy difference between these two states. The energy of the CS state was taken from the temperature dependence emission fittings (Supplementary Notes 5), and the CS state was approximated to be constant throughout the series. Note, that which of S_1 or CS states that is the lowest in energy state in the series varies. Further, the rate for the exothermic direction was assumed to be $3.3 \cdot 10^{10}$ s^{-1} (for the monomer as well as all oligomers), which is the value received from fitting the temperature dependent emission of the monomer (Supplementary Notes 5). The rate constant k_{CStoT1} was assumed to be the same throughout the series. Its value was taken from the monomer, which have a very high quantum yield of ISC. The decay from the CS state is therefore assumed to be dominated by k_{CStoT1} (for the monomer) and could thus be approximated by QY_{ISC}/τ_{CS}. The rate of k_{CStoS0} is in principle what is left in order to explain the excited state lifetime, thus assumed to be $1/\tau_{CS}$ - k_{CStoT1}. The values used for the rates are summarized in Supplementary Table 6 and Supplementary Table 7.

Supplementary Note 8: Analysing the CS recombination using fs-transient absorption and Marcus theory.

To gain more insight into the short CS lifetimes of oligomers in ACN compared to the monomer, femtosecond transient absorption (fsTA) and Marcus theory were employed. The monomer and dimer were analysed in DCM and ACN in order to find causes for the drop in CS lifetime. The fsTA was primarily used to examine if any fast phenomenon had been missed by the ps-ns time resolved emission experiments. Supplementary Figure 42a shows the fsTA spectra of the monomer in ACN obtained with an excitation wavelength of 390 nm. Qualitatively, the signal discernible immediately following excitation can reasonably be assigned to the BODIPY S_1 state, which rapidly evolves into the CS state. The CS state, in turn, evolves into the T_1 state at later times. By leveraging singular value decomposition (SVD) in conjunction with global analysis using the KiMoPack software[10] and applying the kinetic model illustrated in Supplementary Figure 42b, we were able to derive the species-associated spectra (SAS) shown in Supplementary Figure 42c. Supplementary Figure 42d displays selected kinetic traces at 425 nm, 503 nm, and 567 nm, with experimental data denoted by dots and model-generated data represented by lines. The resulting rate constants can be found in Supplementary Table 8. A similar analysis was performed for the dimer in ACN. The corresponding data is shown in Figures 43a-d and the resulting rate constants from the global analysis can be found in Supplementary Table 8. The applied kinetic model fits the data of both monomer and dimer satisfactorily and the extracted rate constants ($\tau_{CSS,monomer}$ = 1/(k_{CStoT1} + k_{CStoS0}) = 2.97 ns and $\tau_{CSS,dimer}$ = 1/(k_{CStoT1} + k_{CStoS0}) = 0.52 ns) corroborates well with the CS state lifetime obtained through TCSPC in Table 1 in the main manuscript.

Marcus theory explains how the rate of charge separation and recombination varies with driving force. The oligomers show a minor stabilization of the CS state of the dimer compared to the monomer (Supplementary Notes 6). Specifically, the slightly lower CS state energy of the dimer could result in a larger rate constant for charge recombination (CR) to the ground state if the CR to the ground state is in the so-called Marcus inverted region. Conversely, if CR to the triplet state is within the Marcus normal region, a slightly lower CS state energy would yield a reduced driving force (since the T_1 energy is approximately the same for both the monomer and the dimer), thus resulting in a smaller rate constant for triplet formation. To validate this hypothesis, the rate constants obtained from the fsTA measurements were compared to Marcus theory.

The rate constant of electron transfer can be calculated using the well-established semi-empirical expression in equation S24.[11, 12]

$$k_{ET} = \frac{2\pi}{\hbar}\frac{H_{DA}^2}{\sqrt{4\pi\lambda k_b T}}\exp\left(-\frac{(\Delta G^0+\lambda)^2}{(4\lambda k_b T)}\right) \qquad \text{(S24)}$$

Where $\hbar$ is Plank's constant divided by 2π, H_{DA} is the electronic coupling between the donor (D) and the acceptor (A), λ is the reorganization energy, k_b is Boltzmann's constant, T is the temperature, and ΔG^0 is the driving force for the reaction. The reorganization energy is the sum of the "inner sphere" (λ_i) and the "outer sphere" (λ_o) components, $\lambda = \lambda_i + \lambda_o$. However, outer-sphere contributions are often the dominant contribution to the total reorganization energy. Hence, in this case, we estimate the total reorganization energy as λ_o since we are primarily interested in an estimation of where the inverted region and normal regions are located energy wise. The outer sphere reorganization energy can be estimated using equation S25.[13, 14]

$$\lambda_o = \frac{e^2}{4\pi\epsilon_0}\left(\frac{1}{2r_D} - \frac{1}{2r_A} + \frac{1}{R_C}\right)\left(\frac{1}{\epsilon_{op}} - \frac{1}{\epsilon_s}\right) \qquad \text{(S25)}$$

Here, e is the elementary charge, ϵ_0 is vacuum permittivity, r_D is the radii of the donor, r_A is the radii of the acceptor, R_c is the donor-acceptor distance, ϵ_{op} is the solvent optical dielectric constant (estimated as the square of the refractive index in the visible region)[15], and ϵ_s is the solvent refractive index. R_c was estimated from the DFT optimized structure as the center of the anthracene to the center of the BODIPY-moiety as 6.55 Å. Assuming the individual radii, r_D and r_A are touching each other they can be approximated as equal and thus cancels out in S25. The refractive index of ACN is 1.3404 and ϵ_s is 37.5. $\lambda_o \approx \lambda$ is thus equal to 1.16 eV. The electron coupling, H_{DA}, is for simplicity assumed to be the same for both the recombination to the ground and triplet states and is found by solving equation S24 with the calculated reorganization energy from equation S25 and with k_{CStoS0}=6.7×10^7 s^{-1} of the monomer together with the corresponding driving force ΔG^0= -2 eV. The driving force is estimated by subtracting the S_1 energy (based on the absorption spectra) from the CS state energy (based on cyclic voltammetry). H_{DA} can thus be calculated to 0.0013 eV.

Supplementary Figure 44 show the measured rates (from Supplementary Table 8) as well as the calculated ones using equation S25 as a function of driving force. The charge recombination to the triplet excited state is in the normal Marcus regime and the charge recombination to the ground state is in the inverted Marcus regime. The deviation from the quadratic curve for some datapoints is not unexpected considering the assumptions that have been made. However, it should be stressed that we are mainly looking at the general trend here, which clearly shows that even if the estimated value of λ is off by ± 0.4 eV it would still place the charge recombination to ground and triplet excited states in the inverted and normal Marcus regimes, respectively.

Supplementary Figures

Chemical characterization

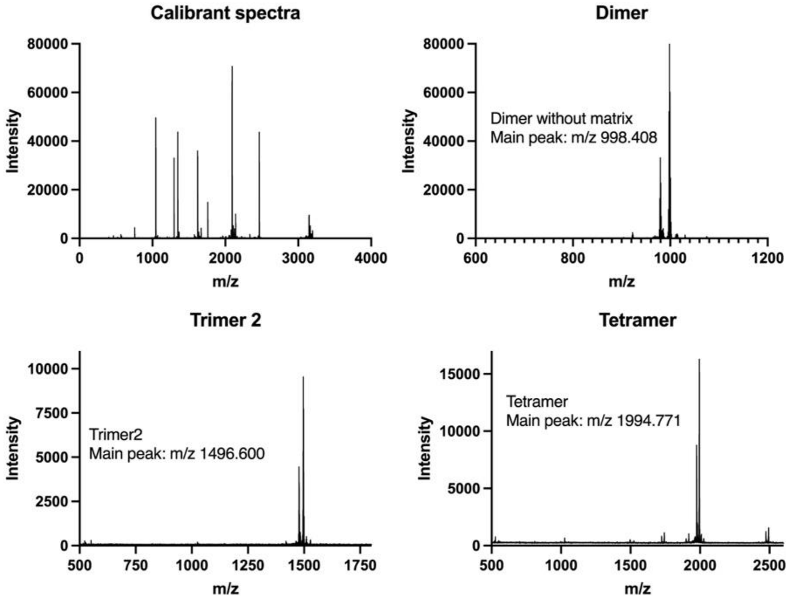

Supplementary Figure 1. MALDI MS: calibrant spectra show mass accuracy below 1ppm. LDI results for the dimer, trimer1, trimer2, and tetramer. All of the compounds were measured without matrix.

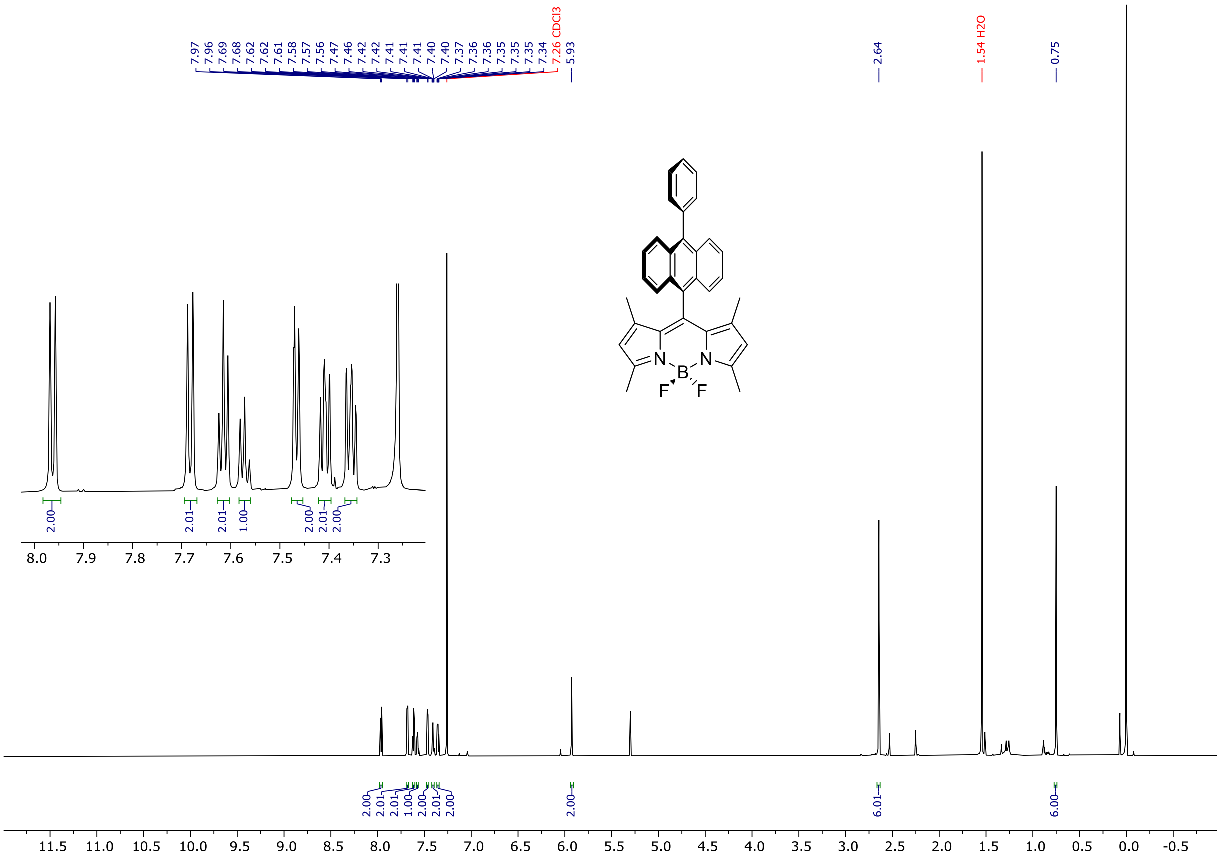

Supplementary Figure 2. ^{1}H (800 MHZ, $CDCl_3$) spectrum of the **phenyl-anthracene-BODIPY dyad**. Analytical HPLC indicate a purity of 98% of this molecule.

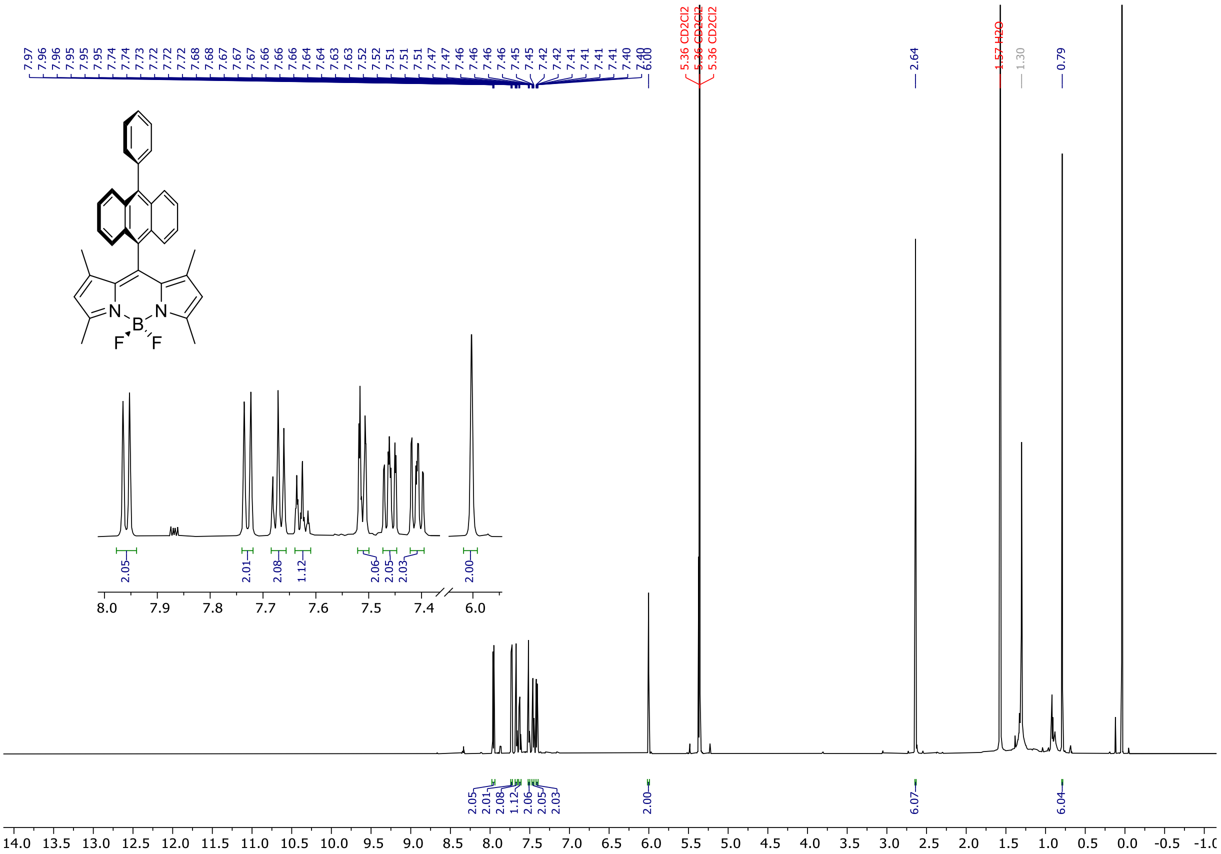

Supplementary Figure 3. ^{1}H (700 MHZ, CD_2Cl_2) spectrum of the **phenyl-anthracene-BODIPY dyad**.

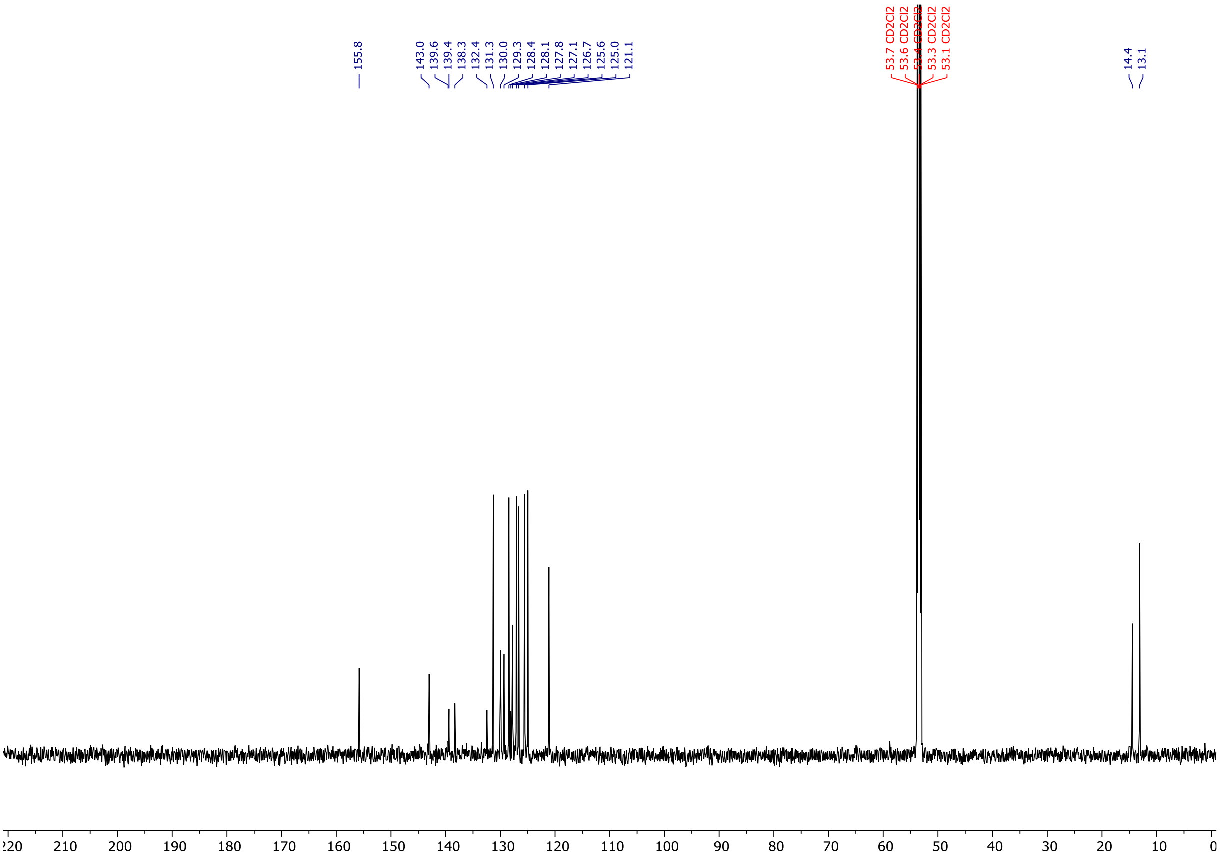

Supplementary Figure 4. ^{13}C (176 MHz, CD_2Cl_2) NMR spectrum of the **phenyl-anthracene-BODIPY dyad**.

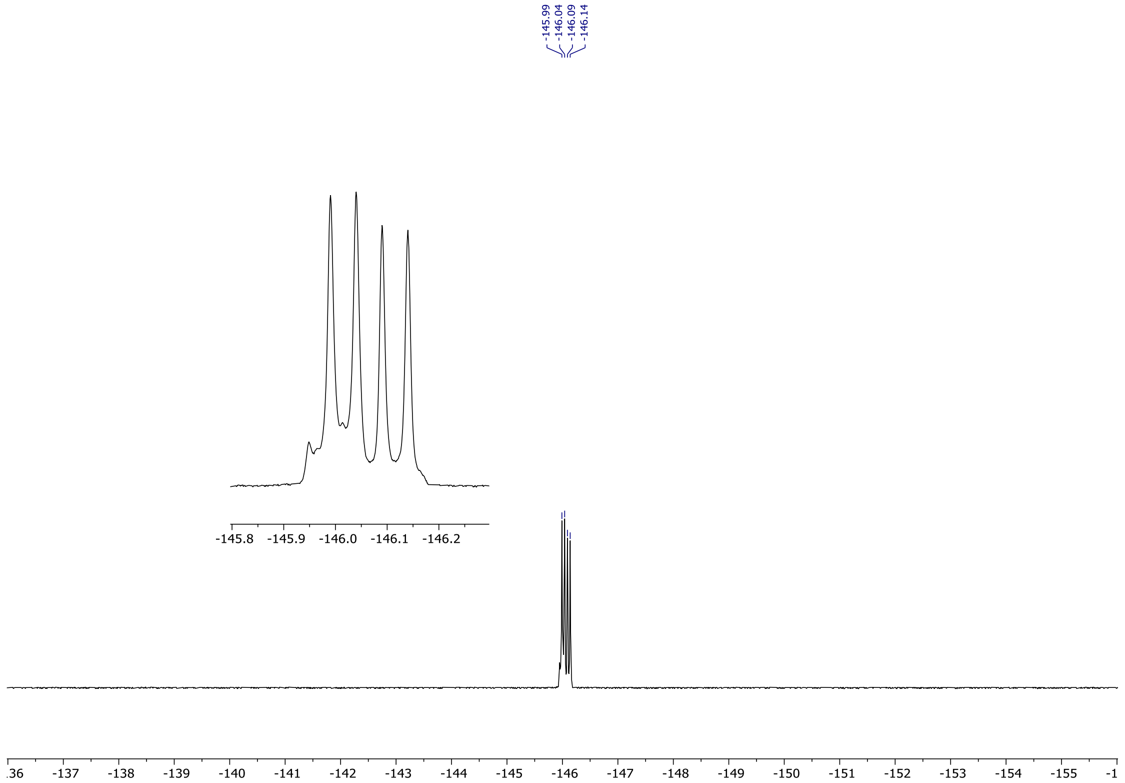

Supplementary Figure 5. ^{19}F NMR (659 MHz, CD_2Cl_2) NMR spectrum of the **phenyl-anthracene-BODIPY dyad.**

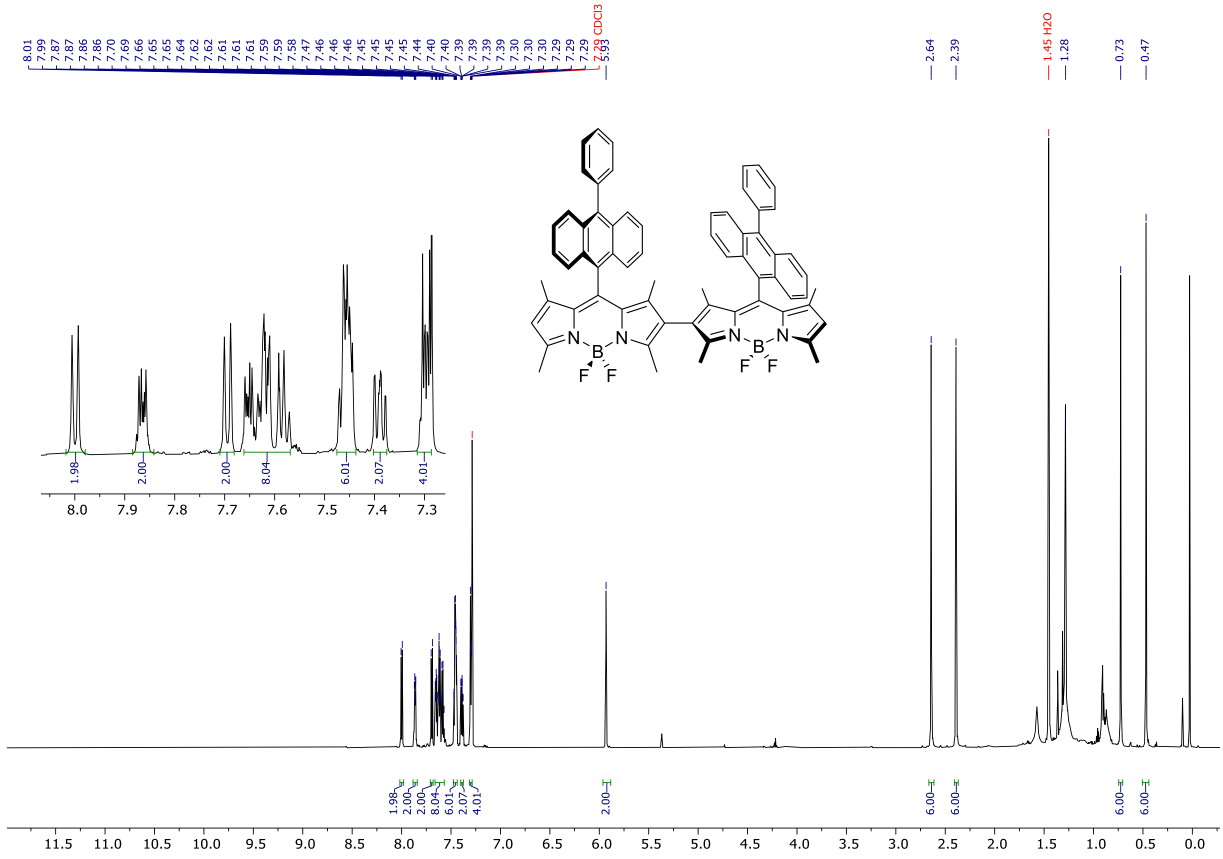

Supplementary Figure 6. ^{1}H (700 MHz, $CDCl_3$) NMR spectrum of the **dimer**. The peak at 1.28 is grease. Analytical HPLC indicate a purity of 97% of this molecule.

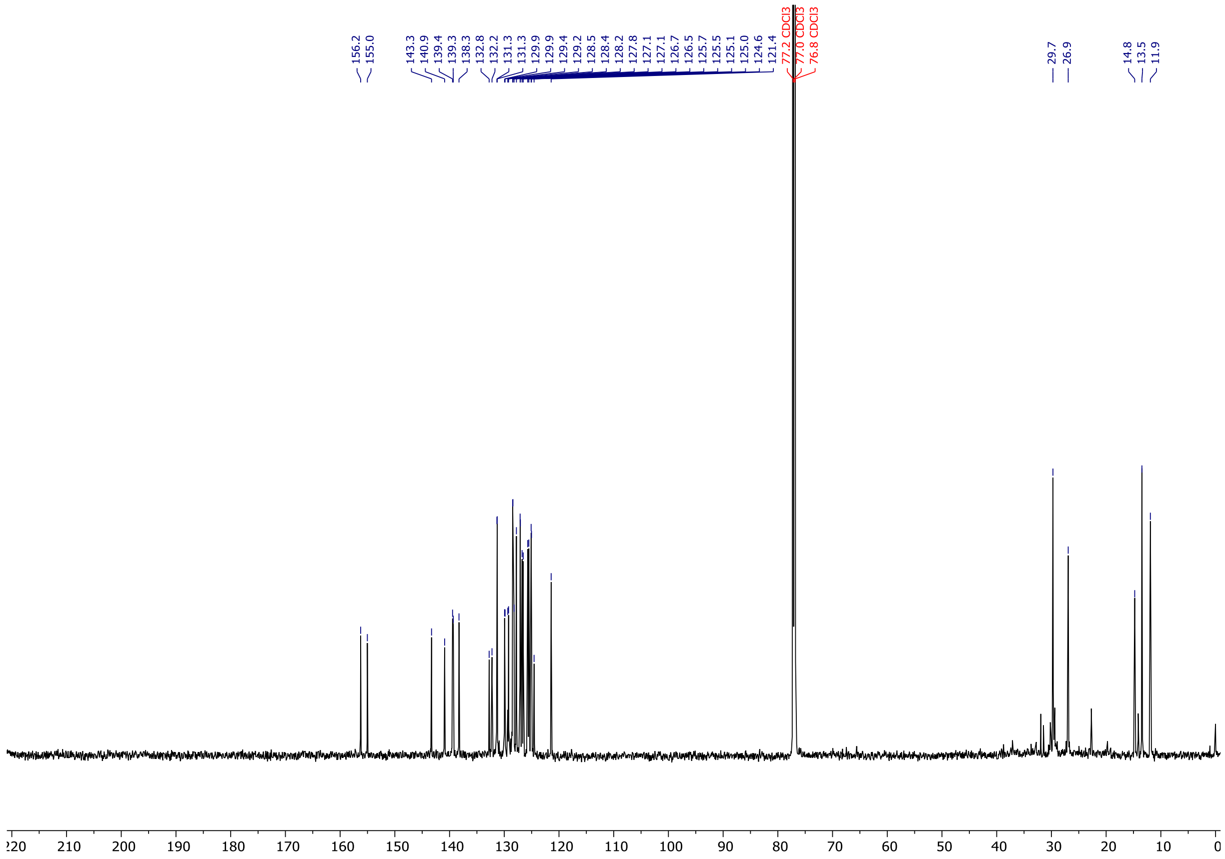

Supplementary Figure 7. ^{13}C (176 MHz, $CDCl_3$) spectrum of the **dimer**. The peak at 29.7 is grease.

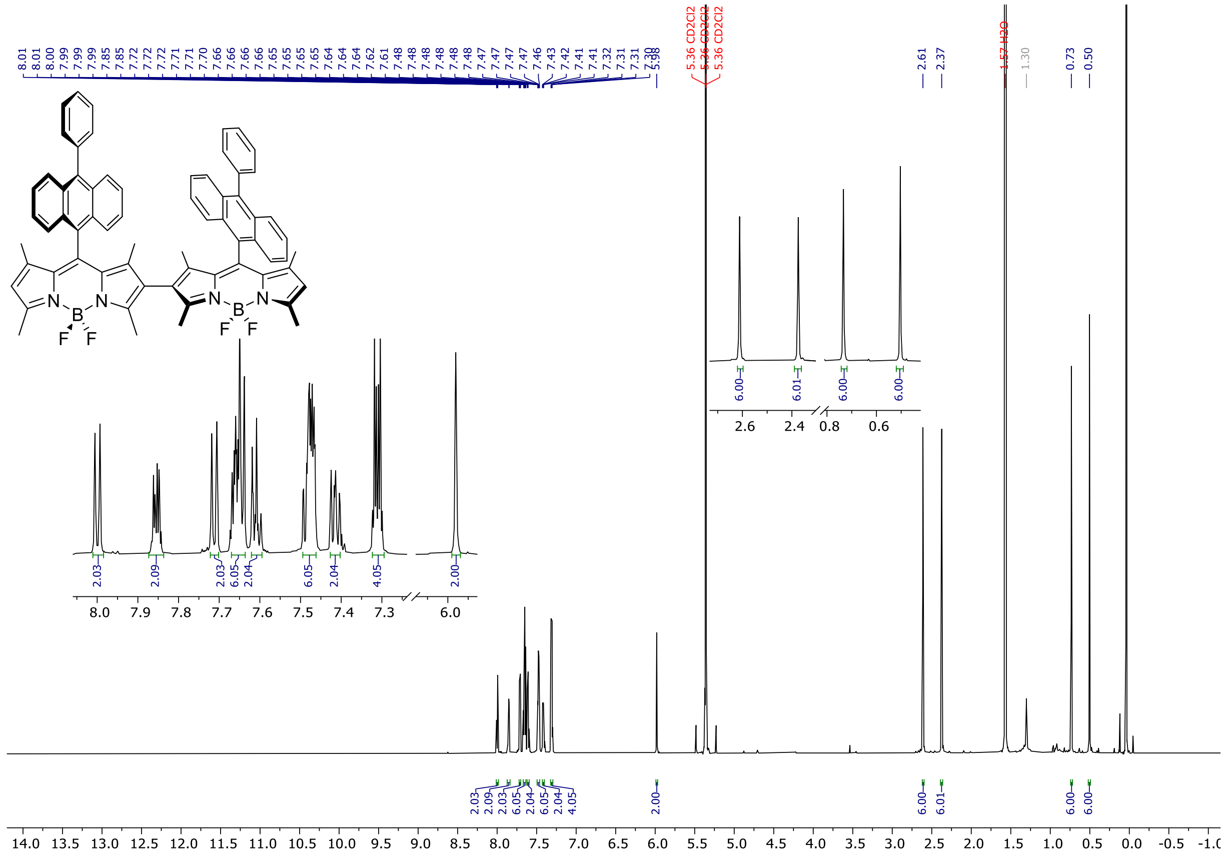

Supplementary Figure 8. ^{1}H (700 MHz, CD_2Cl_2) NMR spectrum of the **dimer**. The peak at 1.30 is grease.

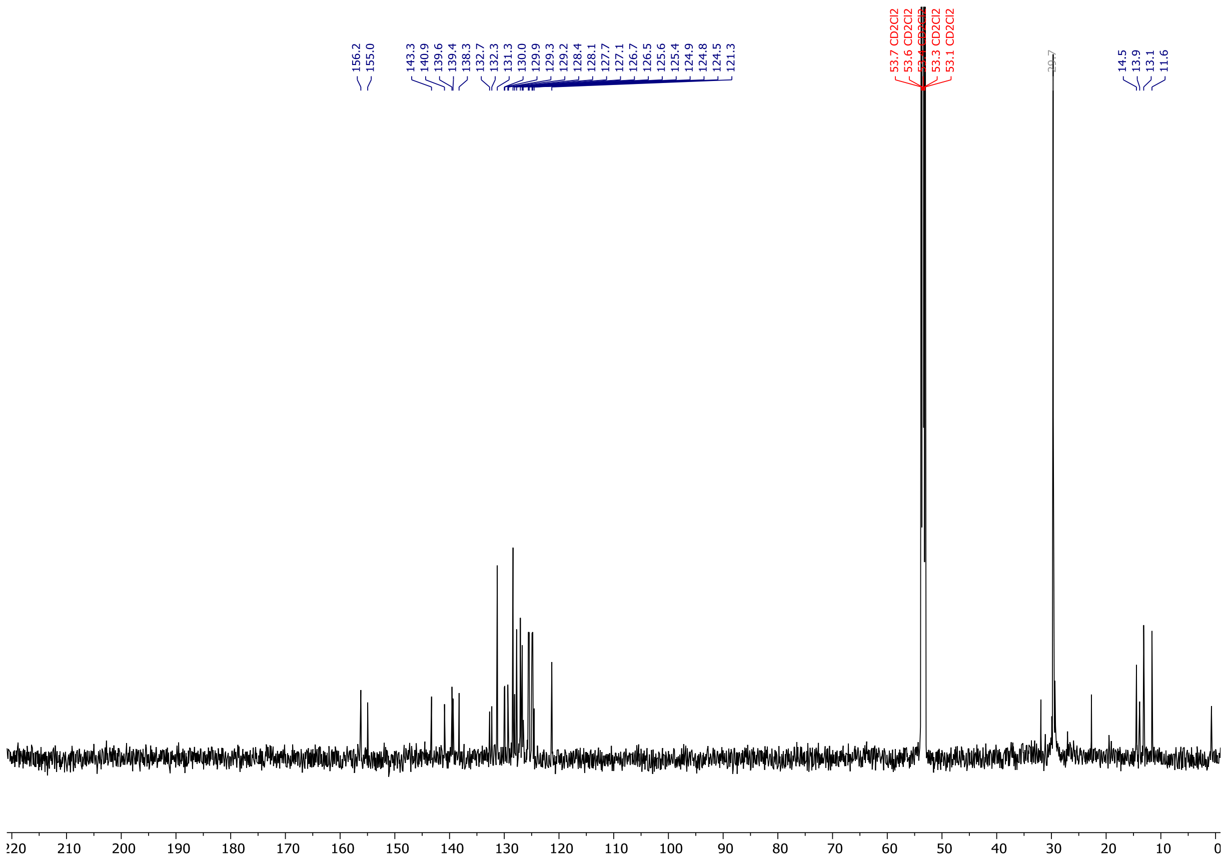

Supplementary Figure 9. ^{13}C (176 MHz, CD_2Cl_2) NMR spectrum of the **dimer**. The peak at 29.7 is grease.

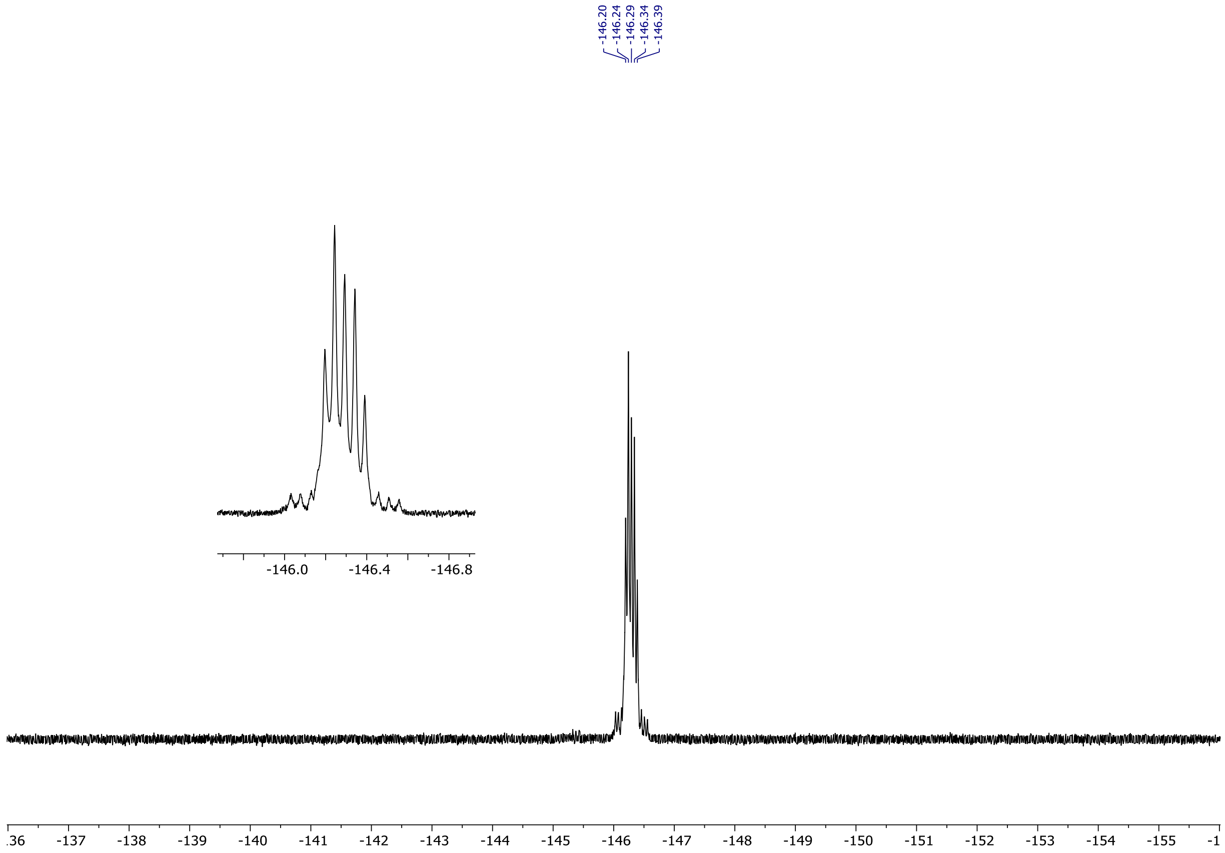

Supplementary Figure 10. ^{19}F NMR (659 MHz, CD_2Cl_2) NMR spectrum of the **dimer**.

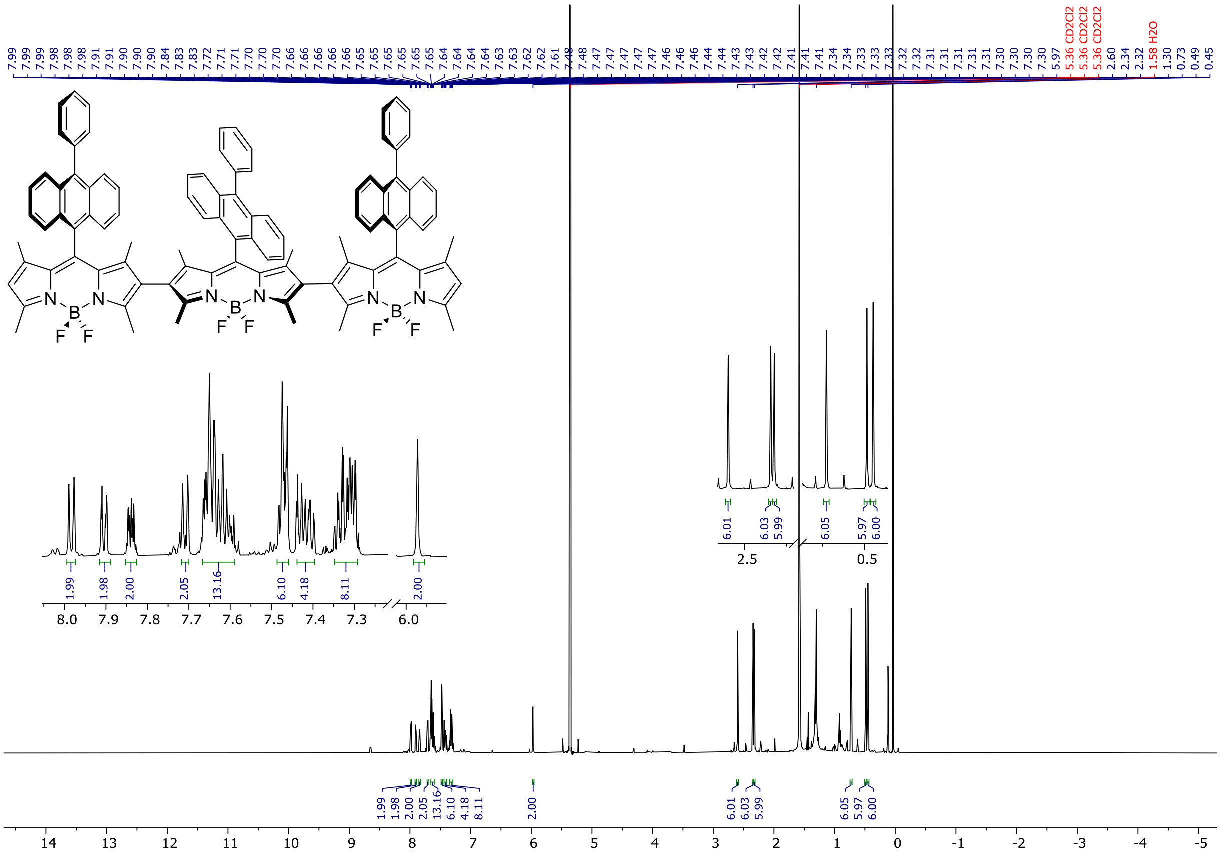

Supplementary Figure 11. ^{1}H (700 MHz, CD_2Cl_2) NMR spectrum of the **trimer 1**. The peak at 1.30 is grease. Analytical HPLC indicate a purity of 95% of this molecule.

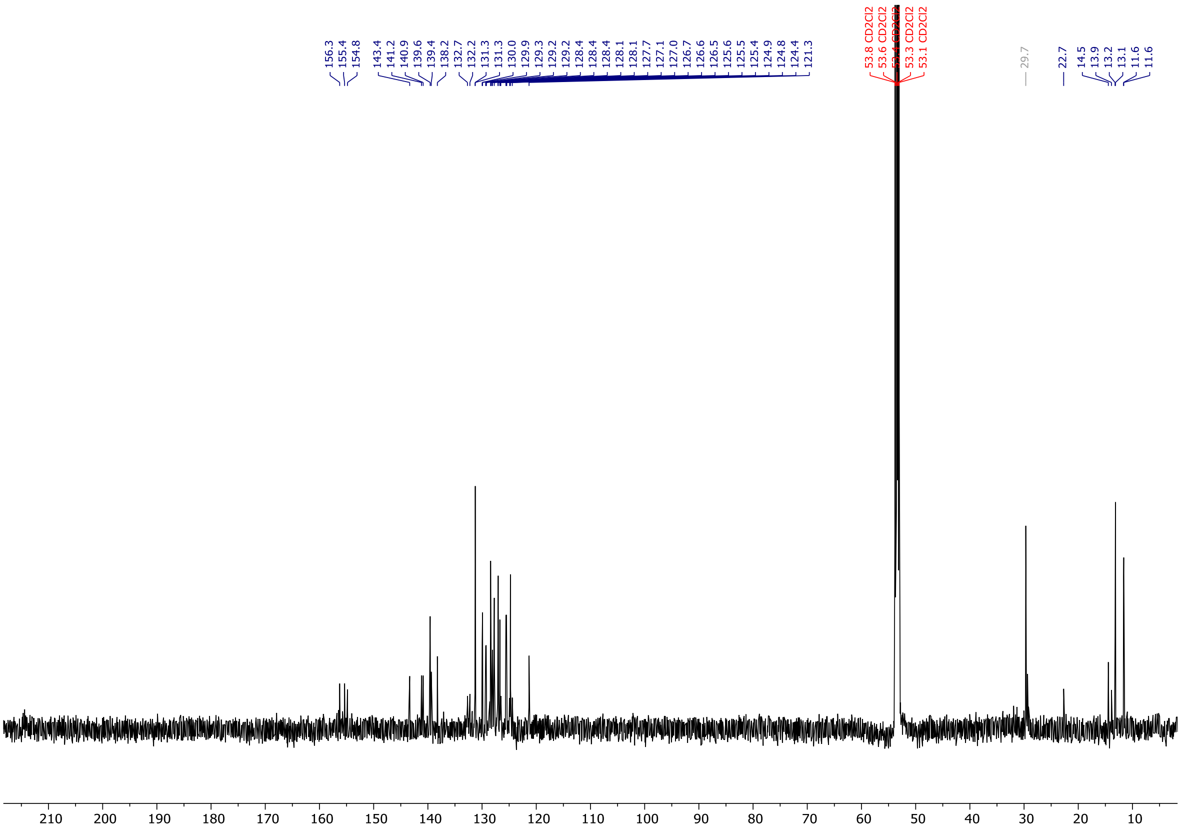

Supplementary Figure 12. ^{13}C (151 MHz, CD_2Cl_2) NMR spectrum of the **trimer 1**. The peak at 29.7 is grease.

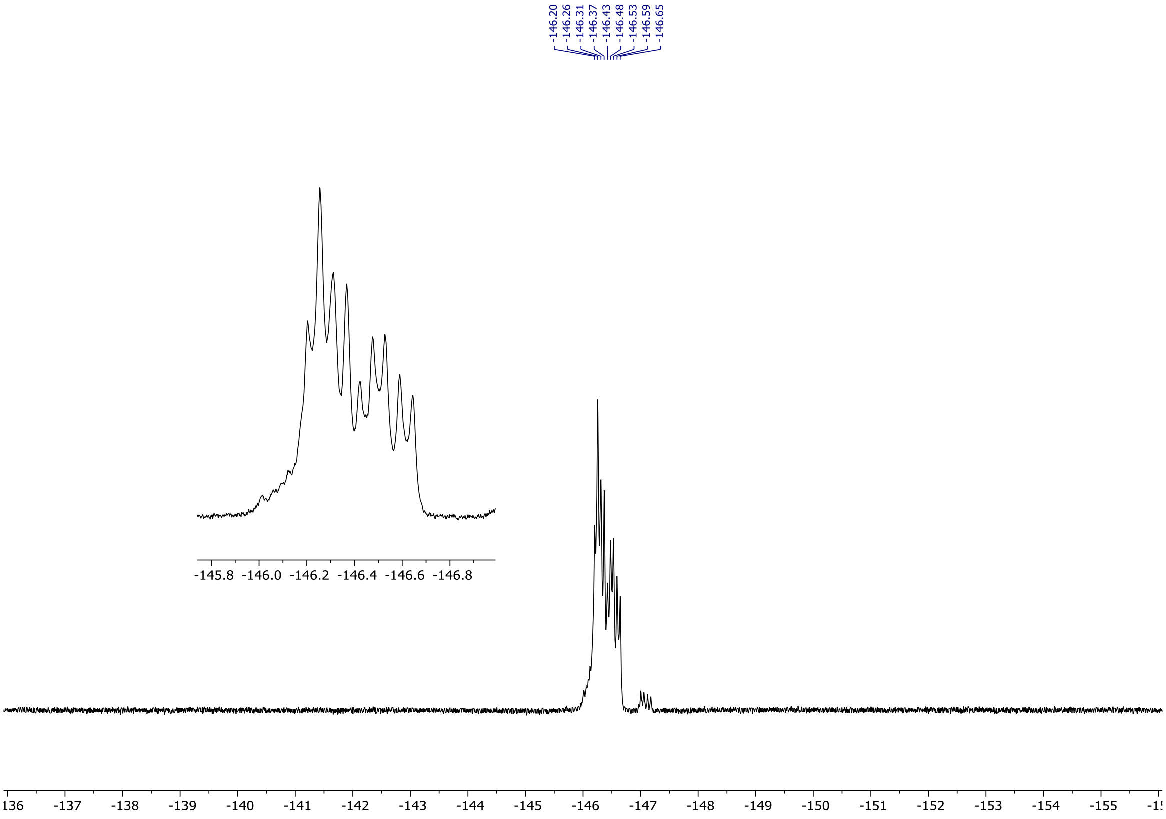

Supplementary Figure 13. ^{19}F (564 MHz, CD_2Cl_2) of the **trimer 1**.

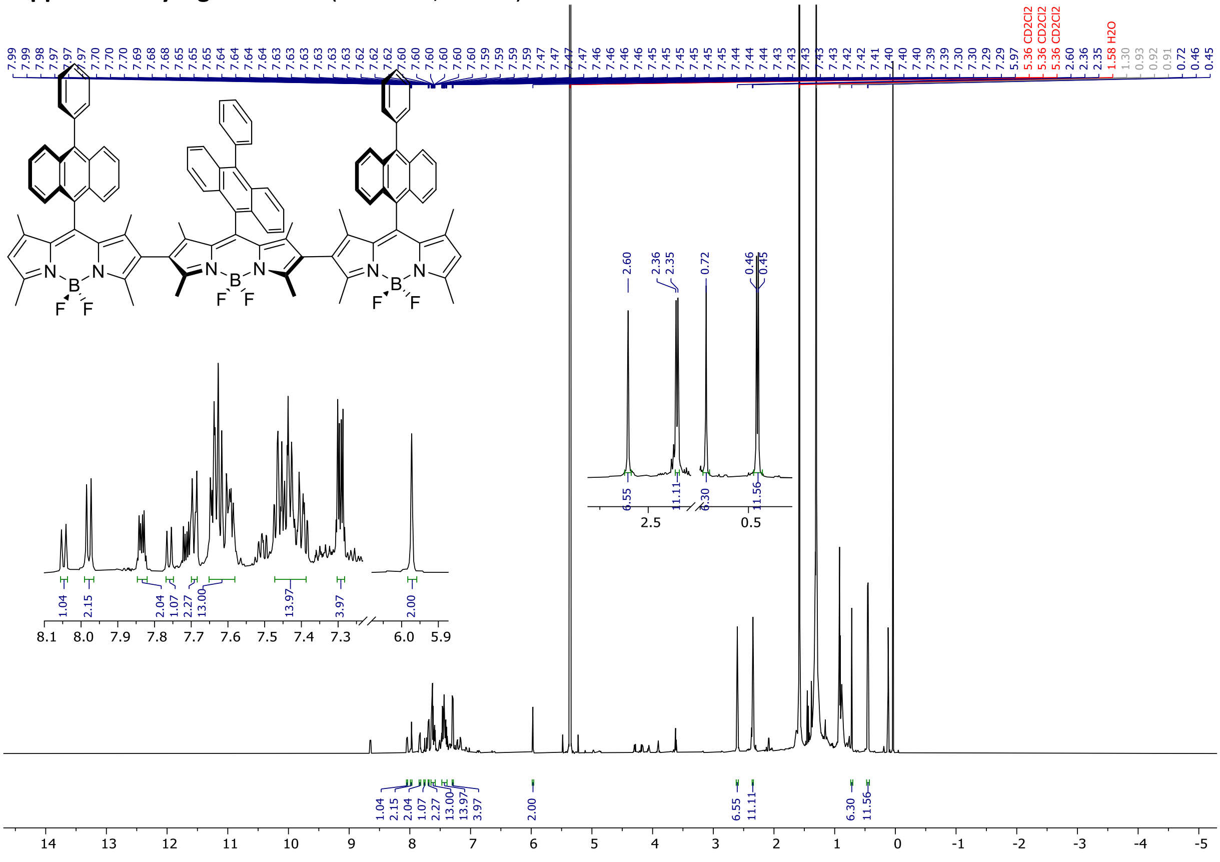

Supplementary Figure 14. ^{1}H (700 MHz, CD_2Cl_2) NMR spectrum of the **trimer 2**. The peak at 1.30 is grease and the triplet at 0.92 belongs to some residual hexane. Analytical HPLC indicate a purity of 98% of this molecule.

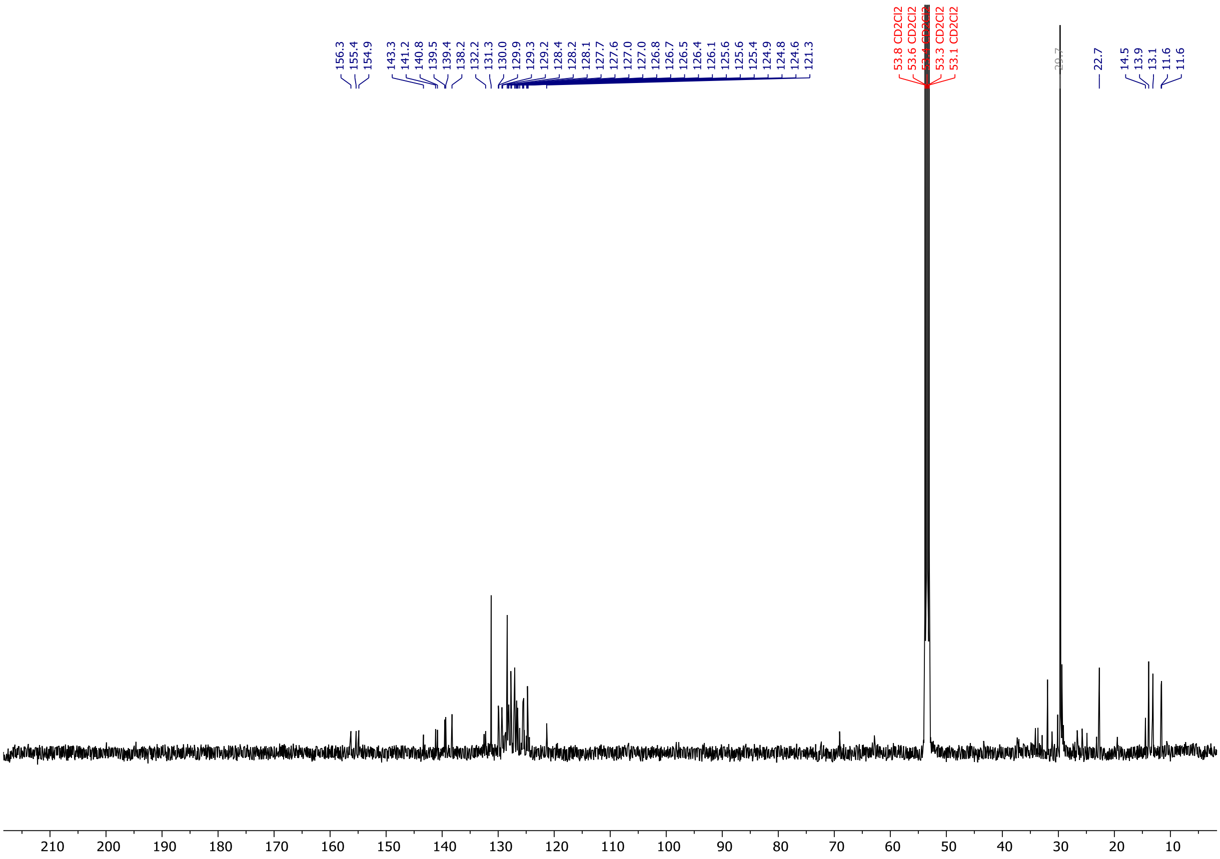

Supplementary Figure 15. ^{13}C (151 MHz, CD_2Cl_2) NMR spectrum of the **trimer 2**. The peak at 29.7 is grease.

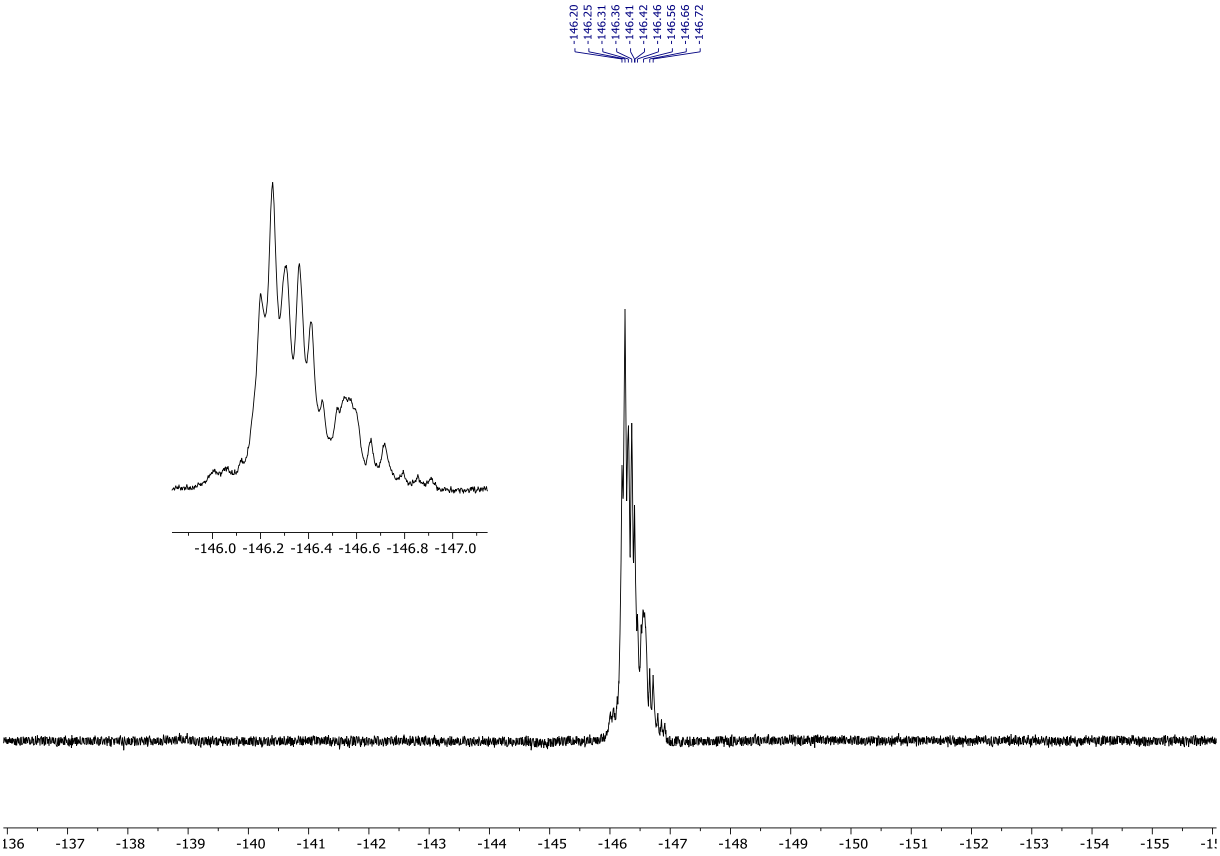

Supplementary Figure 16. ^{19}F (564 MHz, CD_2Cl_2) of the **trimer 2**.

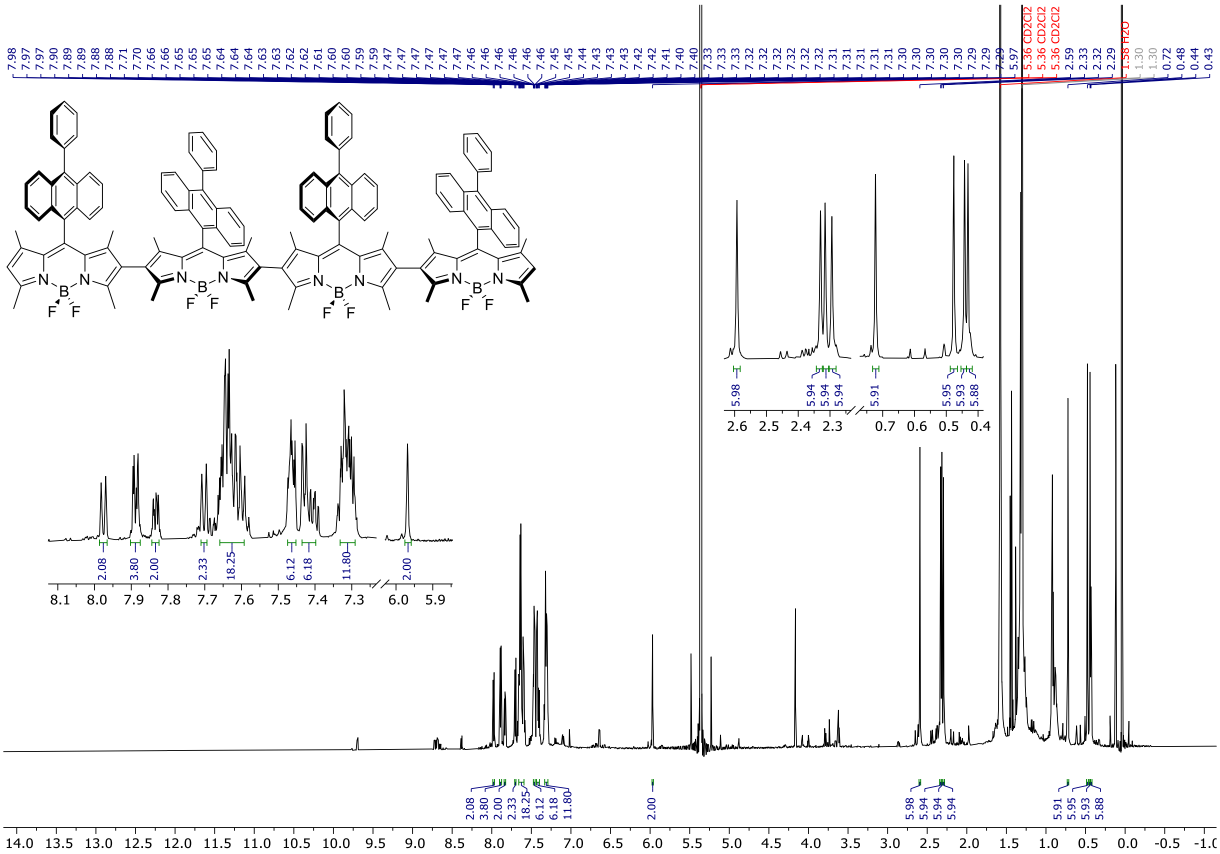

Supplementary Figure 17. ^{1}H (700 MHz, CD_2Cl_2) NMR spectrum of the **tetramer**. The peak at 1.30 is grease. Analytical HPLC indicate a purity of 84% of this molecule.

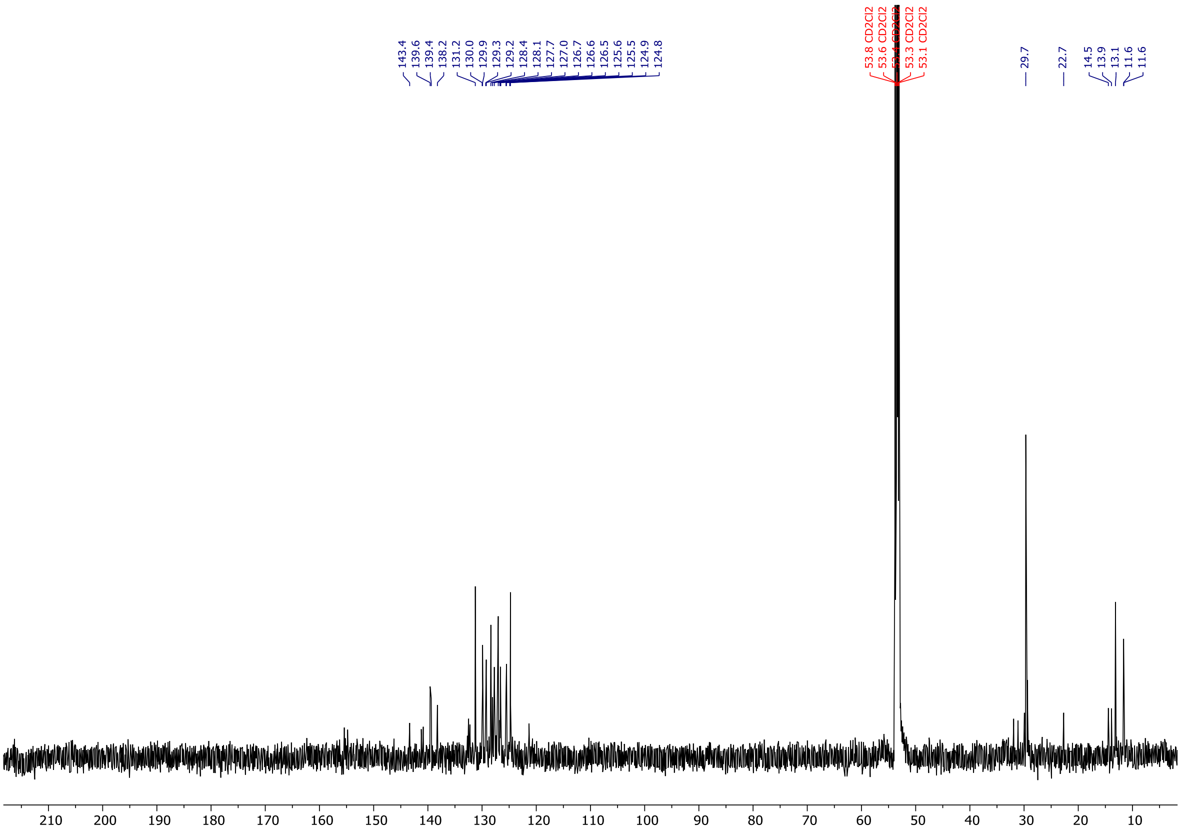

Supplementary Figure 18. ^{13}C (151 MHz, CD_2Cl_2) NMR spectrum of the **tetramer**. The peak at 29.7 is grease.

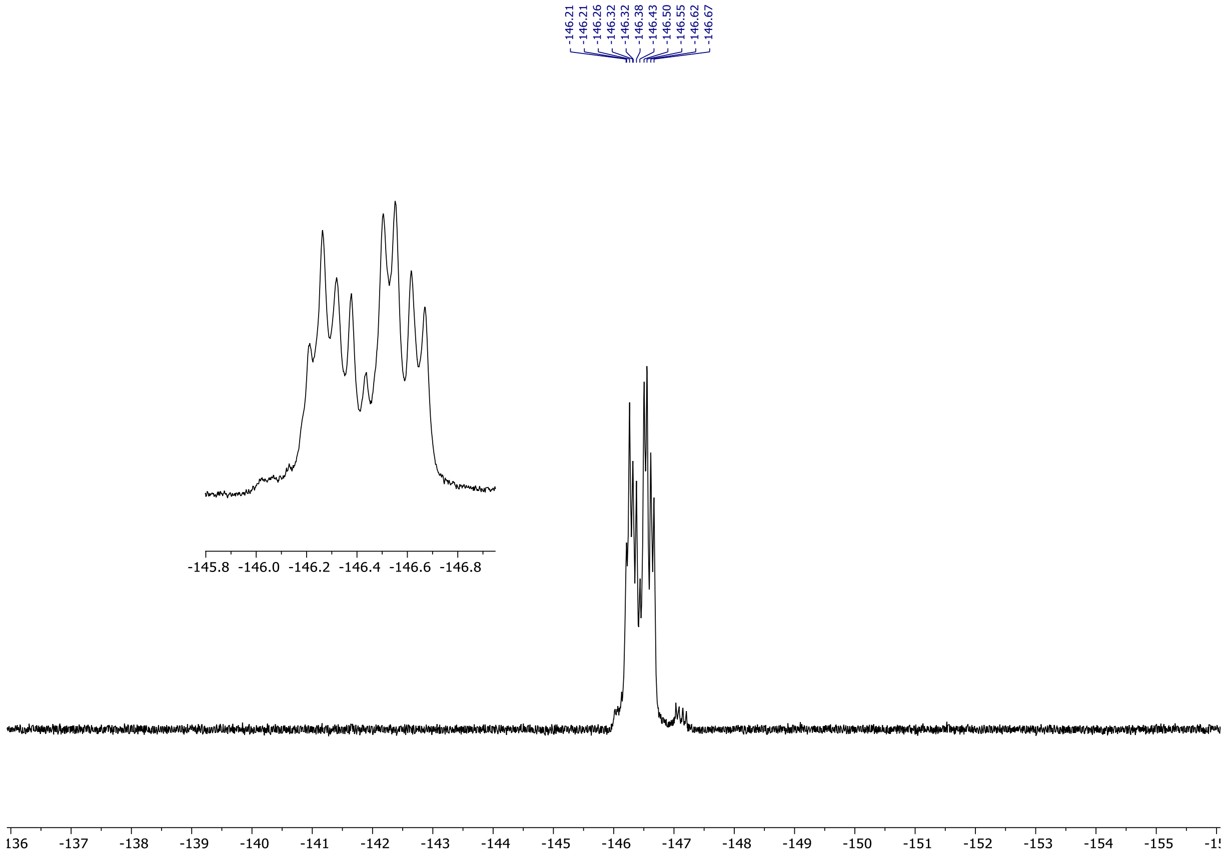

Supplementary Figure 19. ^{19}F (564 MHz, CD_2Cl_2) of the **tetramer.**

Molar absorptivities

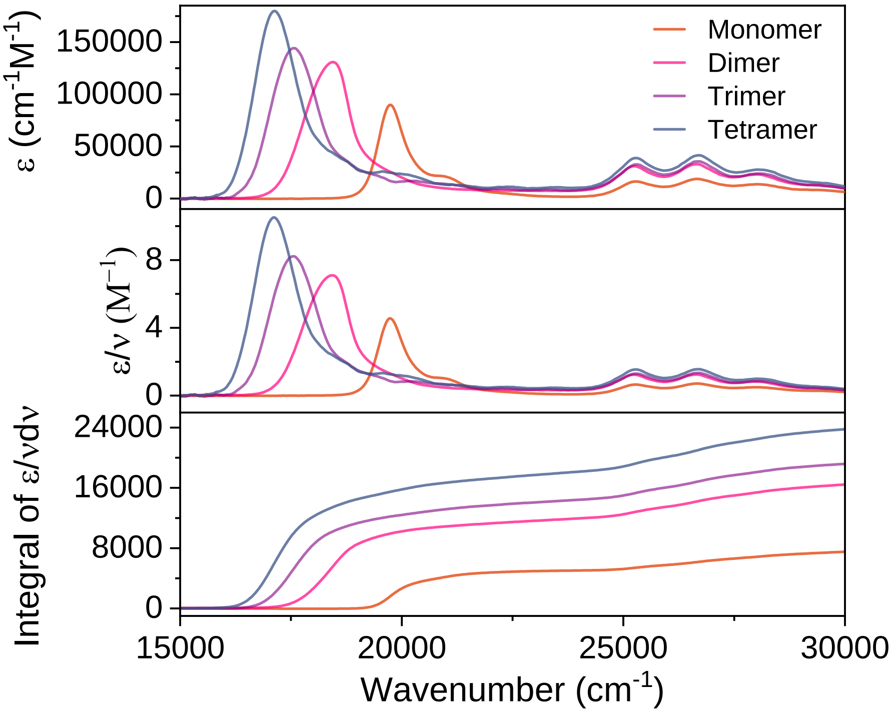

Supplementary Figure 20. Molar absorptivities, molar absorptivities over the wavenumber and the integration thereof for the Monomer, Dimer, Trimer, and Tetramer. The integration was later used to calculate the transition dipole moment using Supplementary Equation 1.

Geometry optimized structures

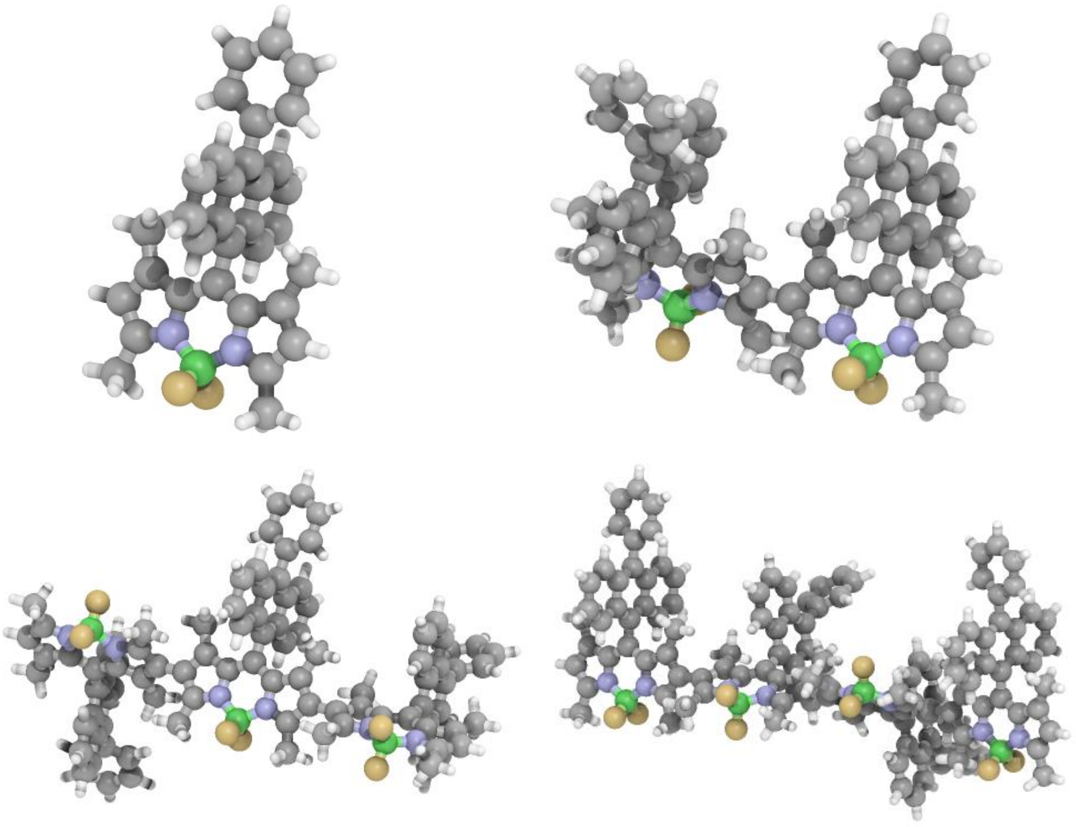

Supplementary Figure 21. Geometry optimized structures on the S_0 surface of the monomer and oligomers using the ωB97XD /6-31g(d) level of theory.

Steady state absorption and emission spectra in various solvents

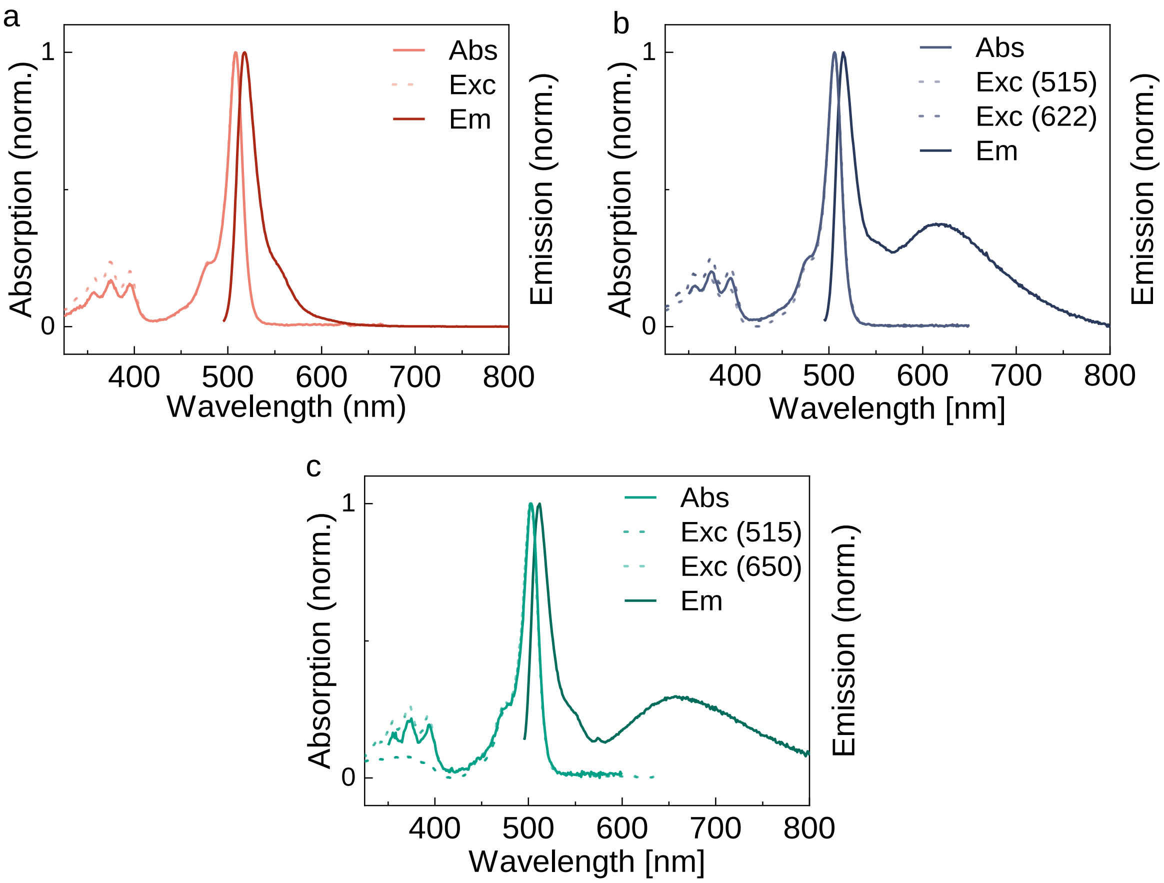

Supplementary Figure 22. Absorption, emission and excitation of the monomer in toluene (a), DCM (b) and ACN (c). Emission were measured upon excitation at 490 nm, where the excitation wavelength are given in the legend. Excitation were measured upon fixed emission at both emission bands, 515 nm and 622 nm in DCM and 515 nm and 650 nm in ACN.

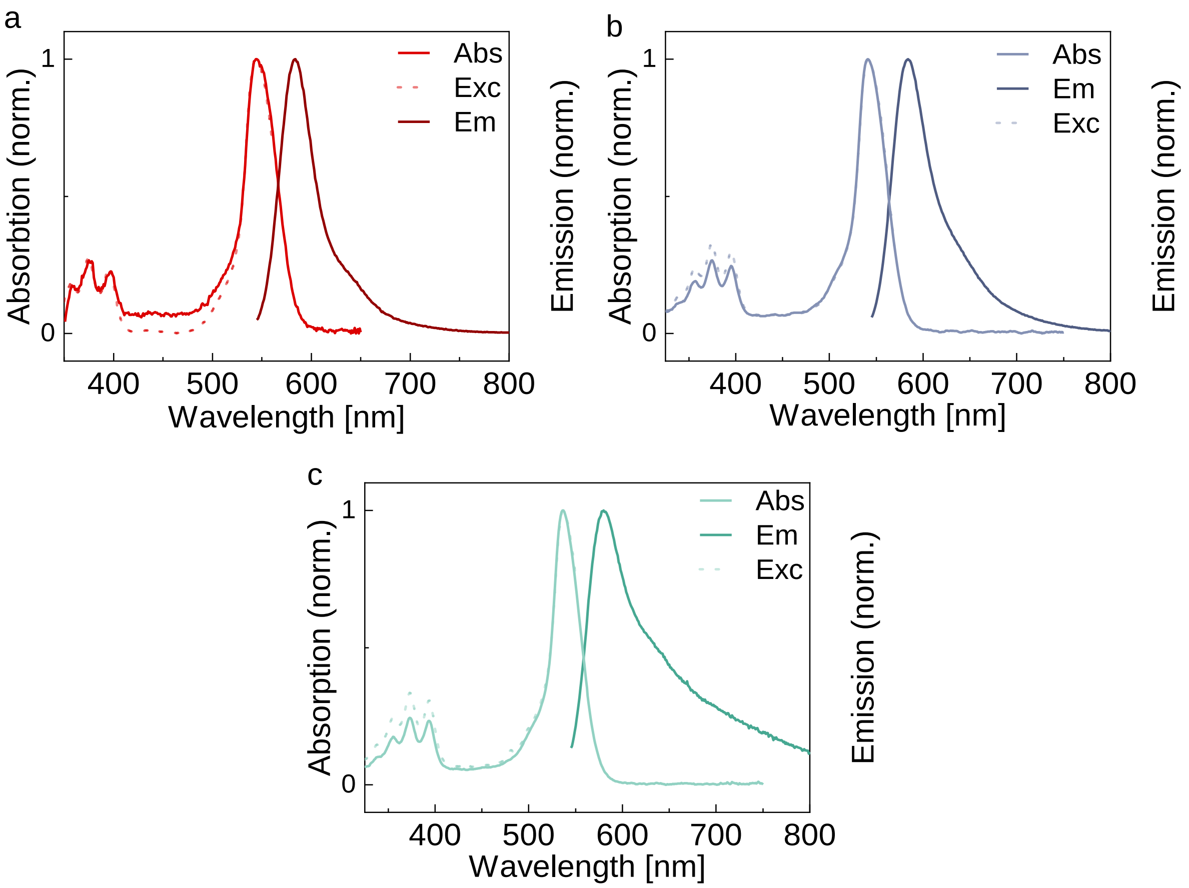

Supplementary Figure 23. Absorption, emission and excitation of the dimer in toluene (a), DCM (b) and ACN (c). Emission were measured upon excitation at 540 nm and excitation were measured with a fixed emission at 580 nm.

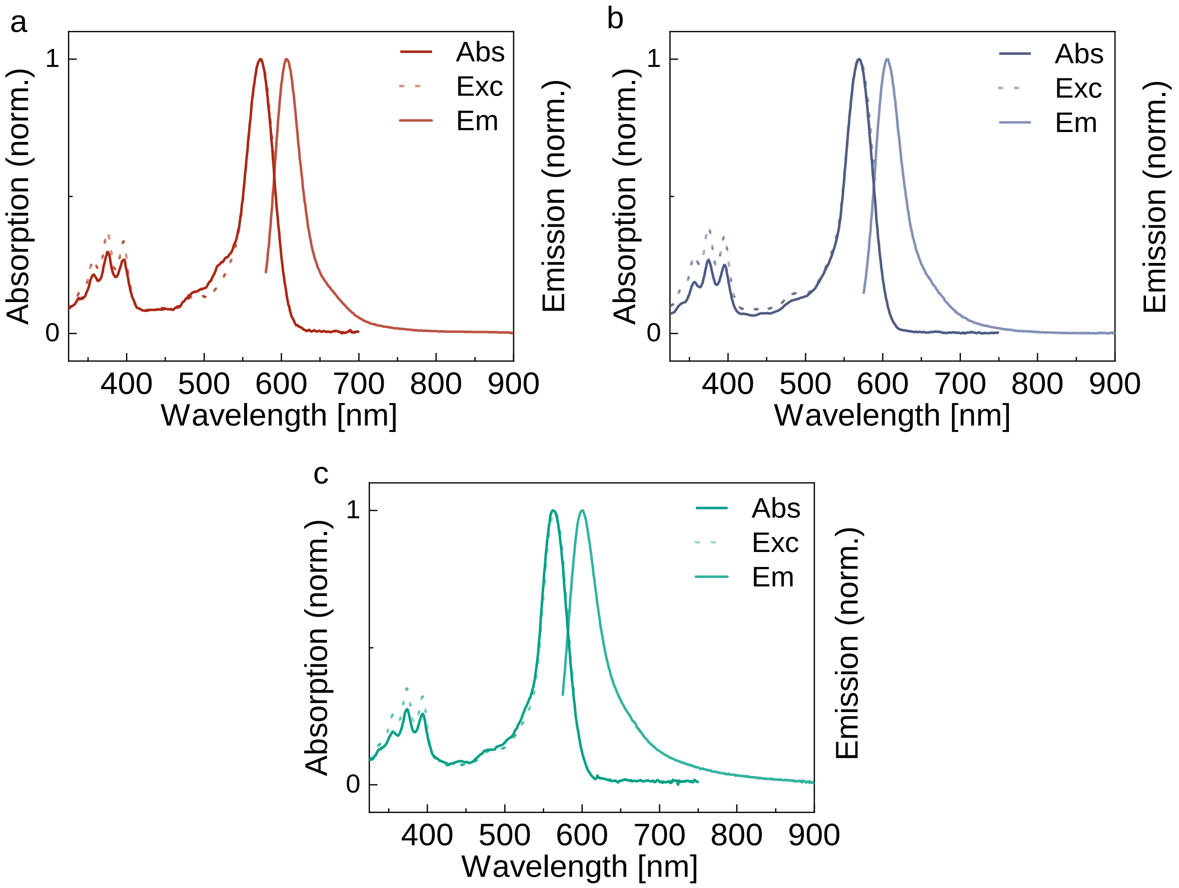

Supplementary Figure 24. Absorption, emission and excitation of trimer 1 in toluene (a), DCM (b) and ACN (c). Emission were measured upon excitation at 570 nm and excitation were measured with a fixed emission at 600 nm.

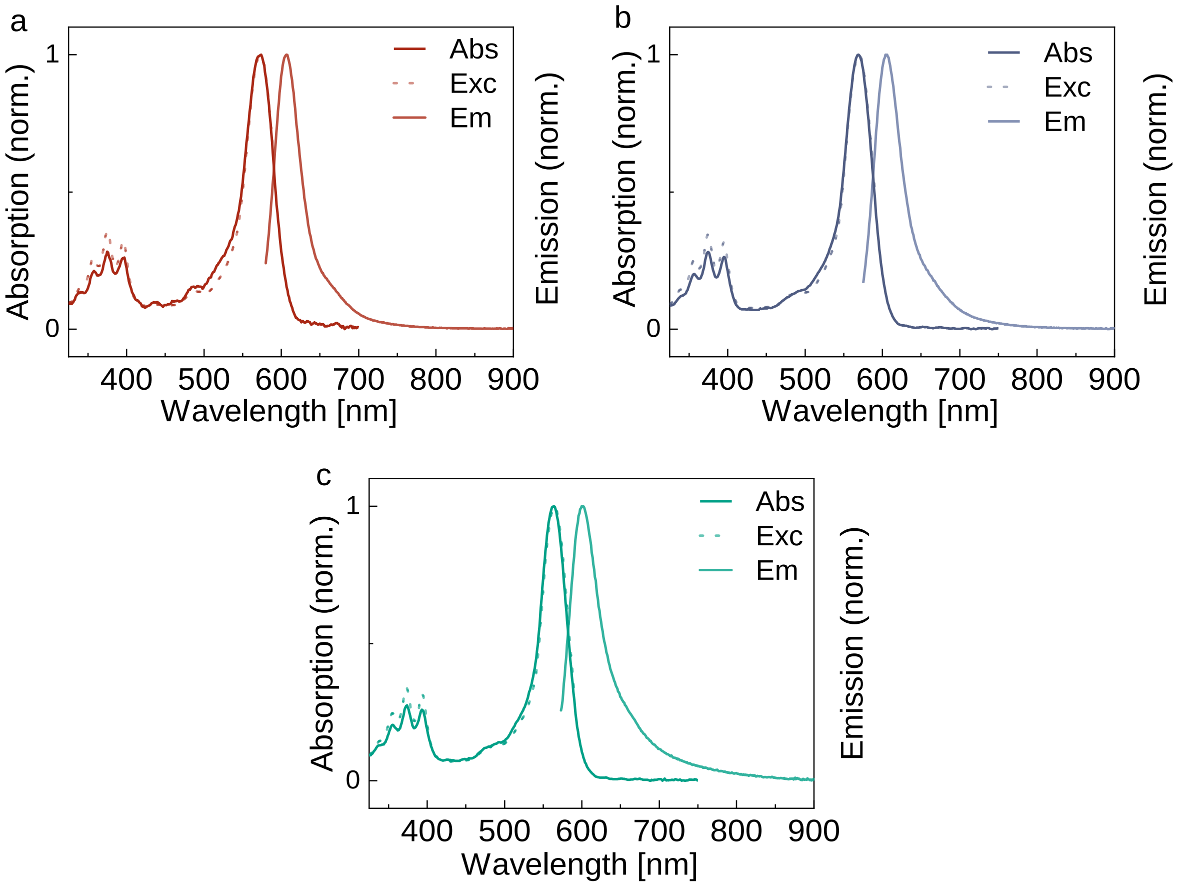

Supplementary Figure 25. Absorption, emission and excitation of trimer 2 in toluene (a), DCM (b) and ACN (c). Emission were measured upon excitation at 570 nm and excitation were measured with a fixed emission at 600 nm.

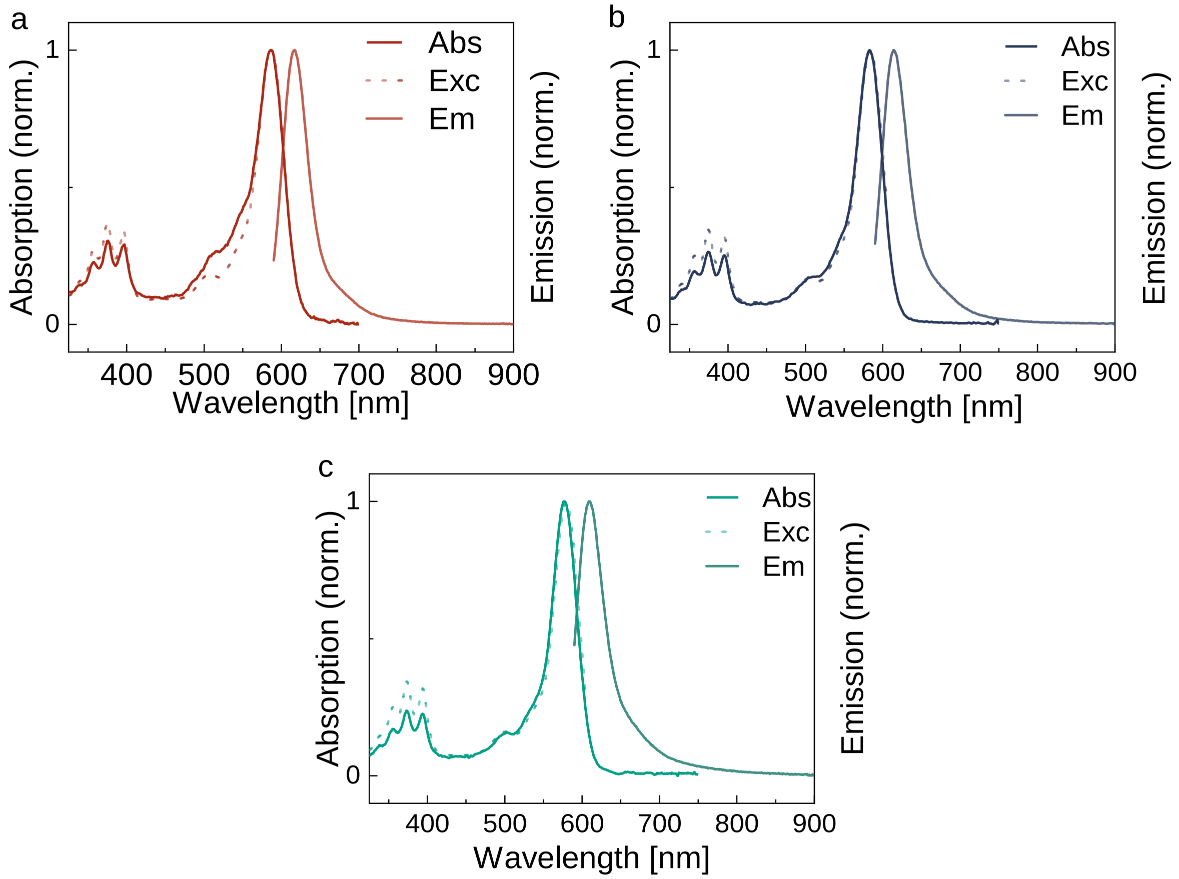

Supplementary Figure 26. Absorption, emission and excitation of the tetramer in toluene (a), DCM (b) and ACN (c). Emission were measured upon excitation at 585 nm and excitation were measured with fixed emission at 615 nm.

Time resolved emission

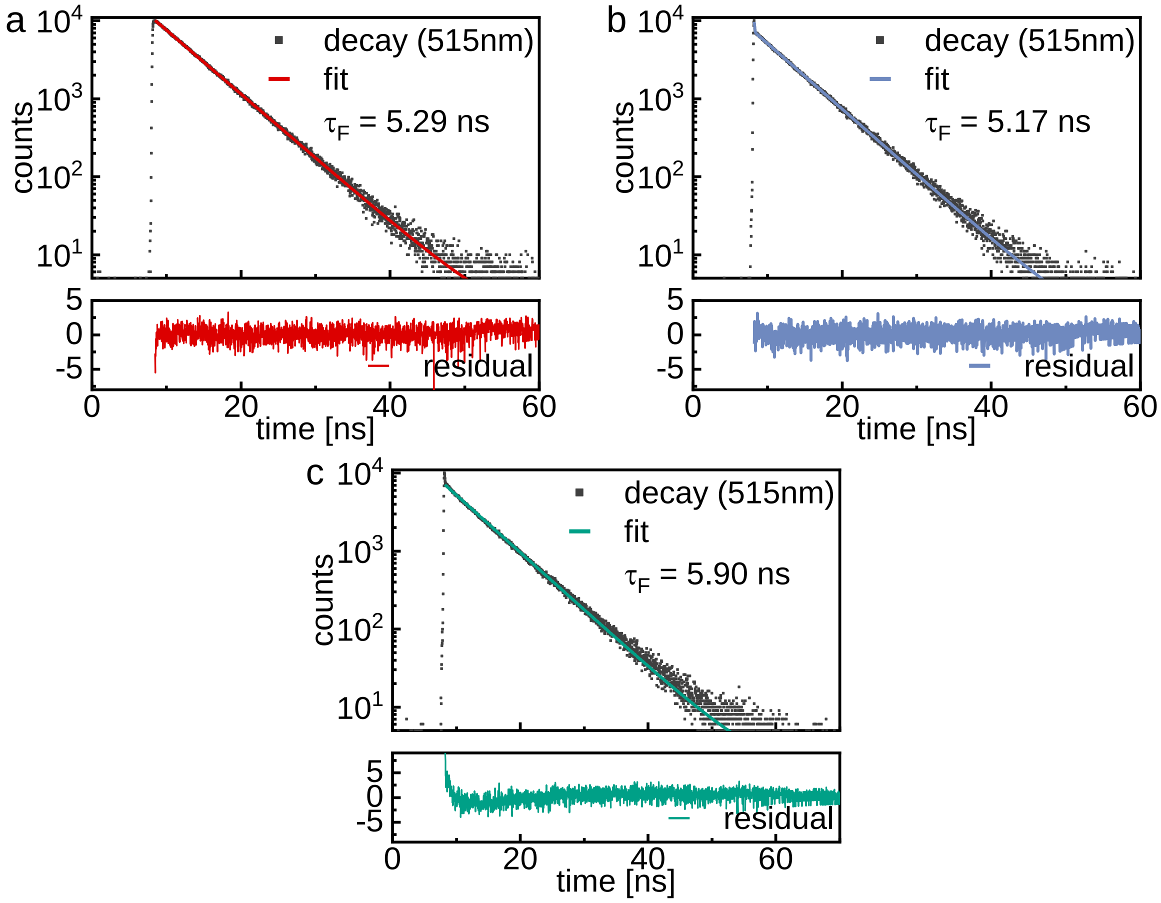

Supplementary Figure 27. Lifetime measurements of the monomer at the BODIPY emission (515 nm) in toluene (a), DCM (b), and ACN (c). Emission lifetime was taken upon excitation at 510 nm. The data was fitted using a tailfit, which is shown as a coloured line in the decays.

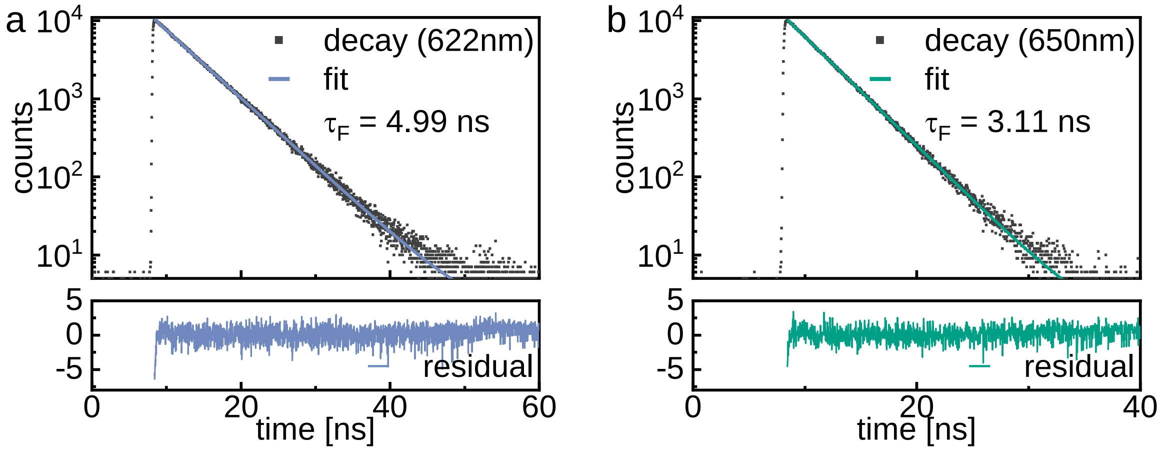

Supplementary Figure 28. Lifetime measurements of the monomer at the second emission band (CS state-emission) in DCM (a) at 622 nm, and ACN (b) at 650 nm. Emission lifetimes were taken upon excitation at 510 nm. The data was fitted using a tailfit, which is shown as a coloured line in the decays.

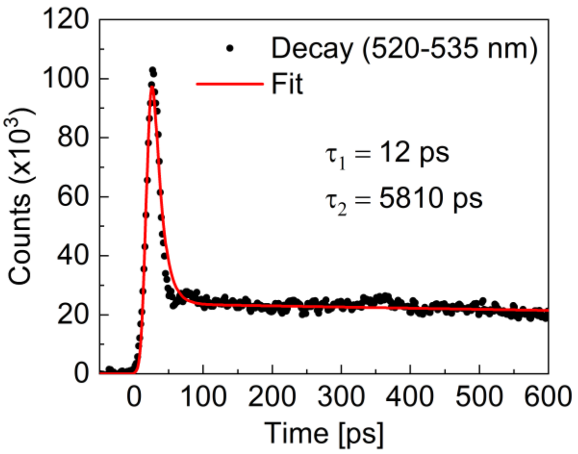

Supplementary Figure 29. Streak camera measurement of the monomer emission (520-535 nm) in DCM. The fast component is barely resolved with a pulse width of 9 ps and the long lifetime of 5.8 ns matches well with the TCSPC data. The red line shows a fit using a fast component that was locked to 12 ps.

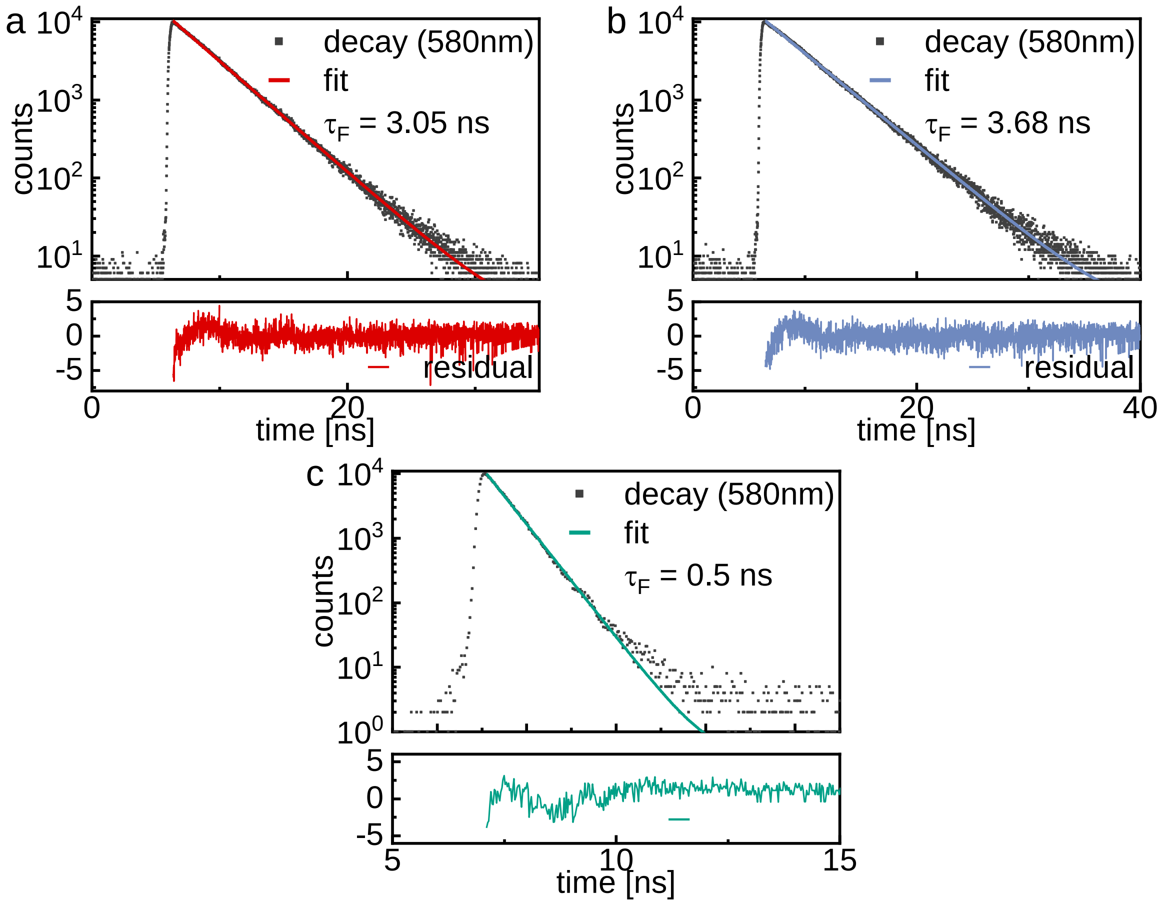

Supplementary Figure 30. Lifetime measurements of the dimer at the BODIPY emission (580 nm) in toluene (a), DCM (b), and ACN (c). Emission lifetimes were taken upon excitation at 375 nm. The data were fitted using a tailfit, which are shown as a coloured line in the decays.

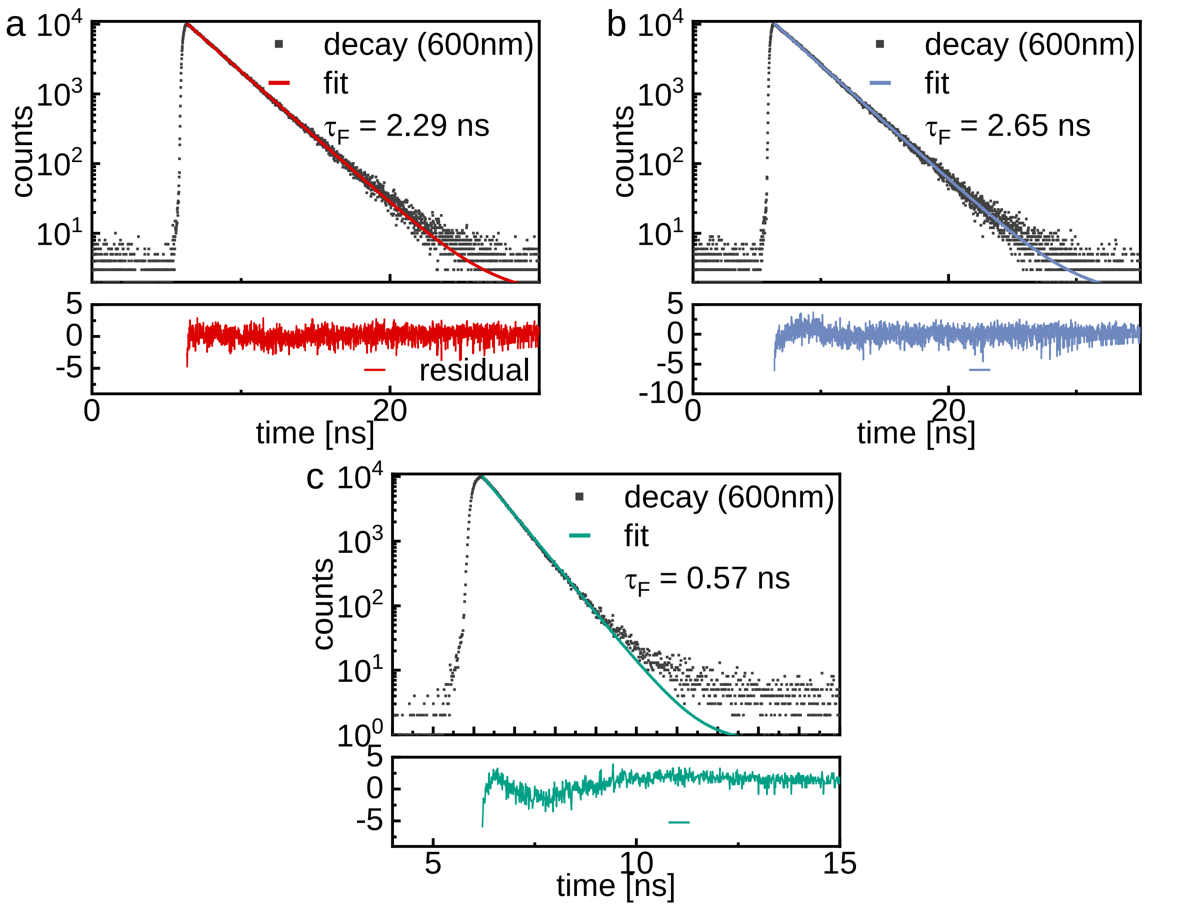

Supplementary Figure 31. Lifetime measurements of trimer 1 at the BODIPY emission (600 nm) in toluene (a), DCM (b), and ACN (c). Emission lifetimes were taken upon excitation at 375 nm. The data were fitted using a tailfit, which are shown as a coloured line in the decays. The lower spectrum shows the residual of the fit.

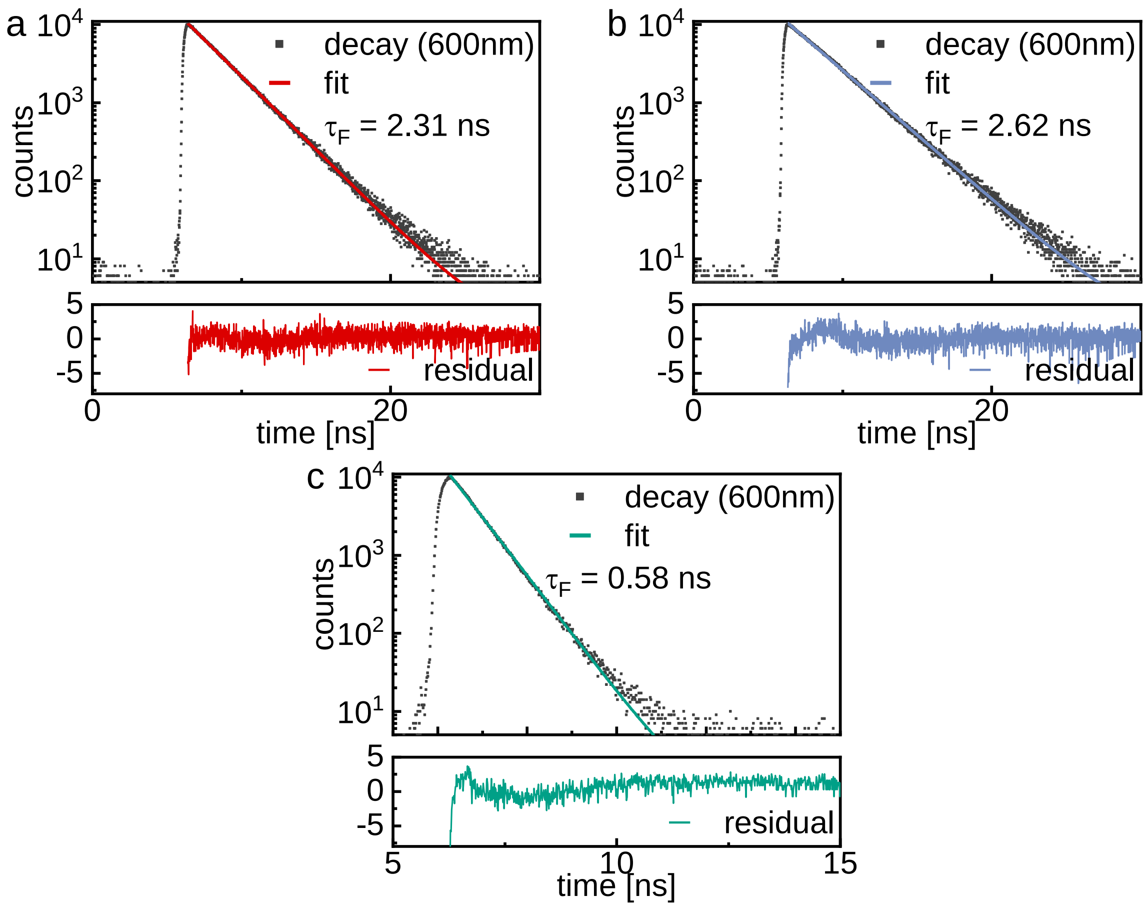

Supplementary Figure 32. Lifetime measurements of trimer 2 at the BODIPY emission (600 nm) in toluene (a), DCM (b), and ACN (c). Emission lifetimes were taken upon excitation at 375 nm. The data were fitted using a tailfit, which are shown as a coloured line in the decays.

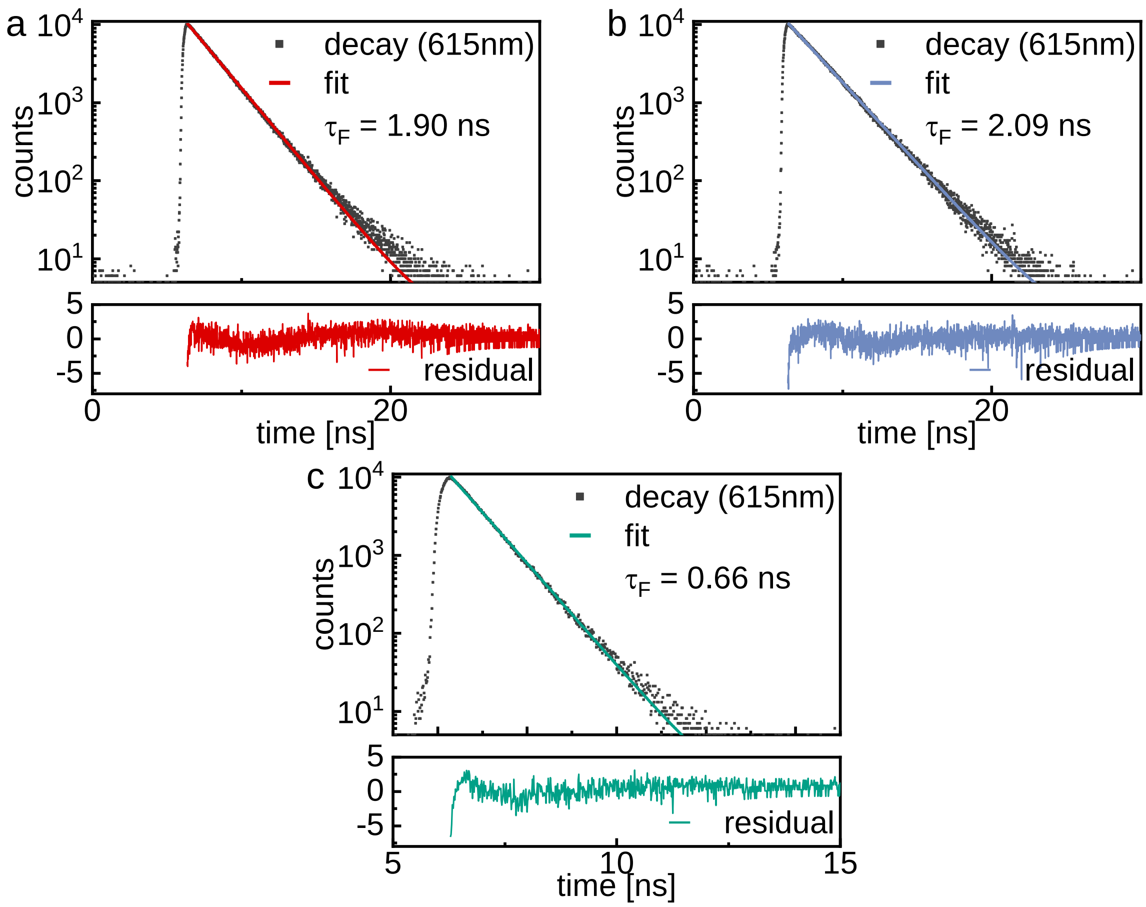

Supplementary Figure 33. Lifetime measurements of the tetramer at the BODIPY emission (615 nm) in toluene (a), DCM (b), and ACN (c). Emission lifetimes were taken upon excitation at 375 nm. The data were fitted using a tailfit, which are shown as a coloured line in the decays.

Temperature dependent emission

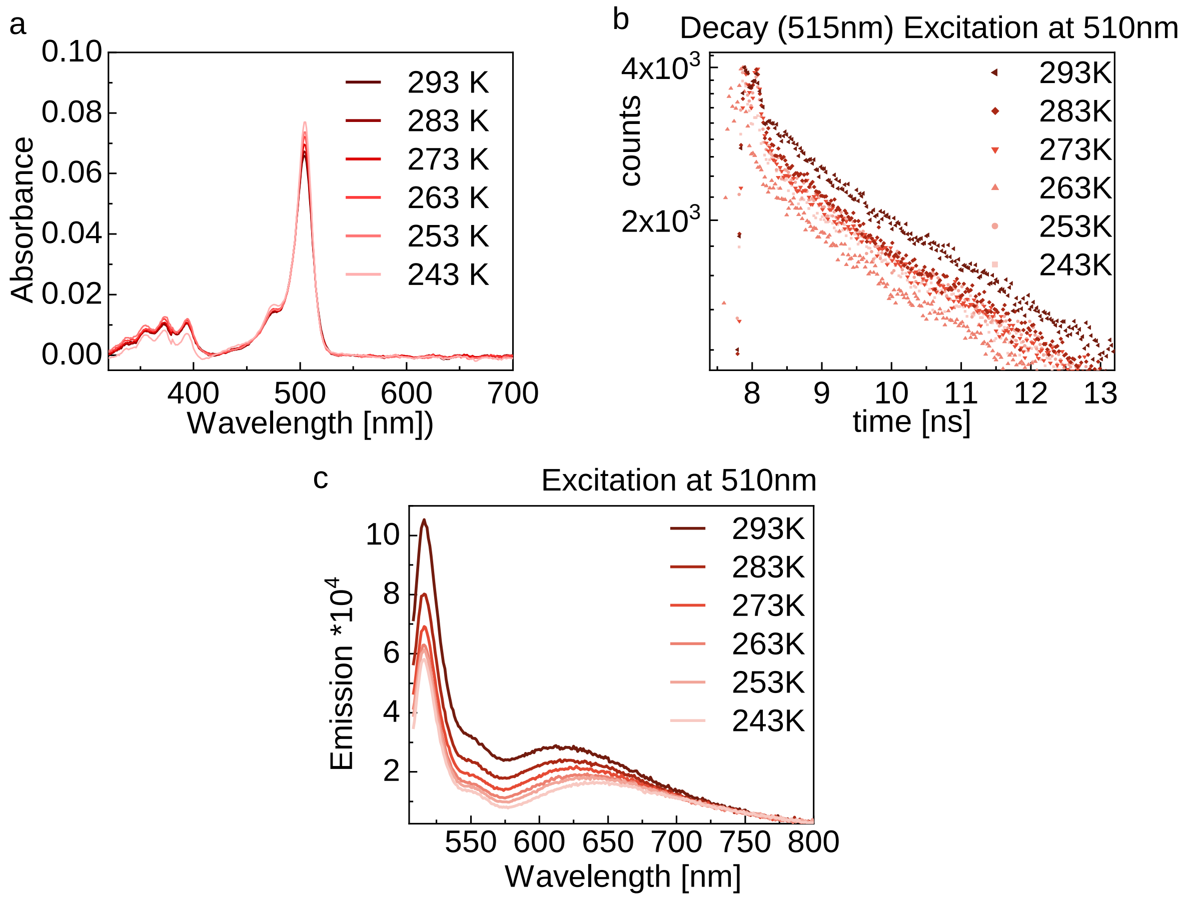

Supplementary Figure 34. Temperature dependent lifetime (a) with a zoom in (b), and steady state emission (c) of the monomer from 293 K to 243 K. The lifetime was recorded upon excitation at 510 nm.

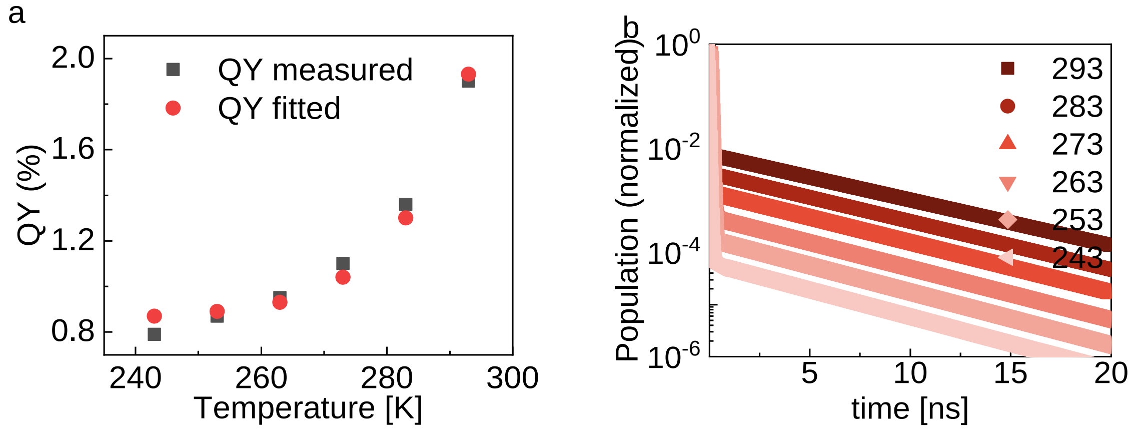

Supplementary Figure 35. (a) Temperature dependent emission excited at 500 nm scaled to the fluorescence quantum yield, and the theoretical fluorescence quantum yields received when fitted to Supplementary Equation 11. (b) Simulated fluorescence decays using Supplementary Equations 8-9.

Electrochemistry

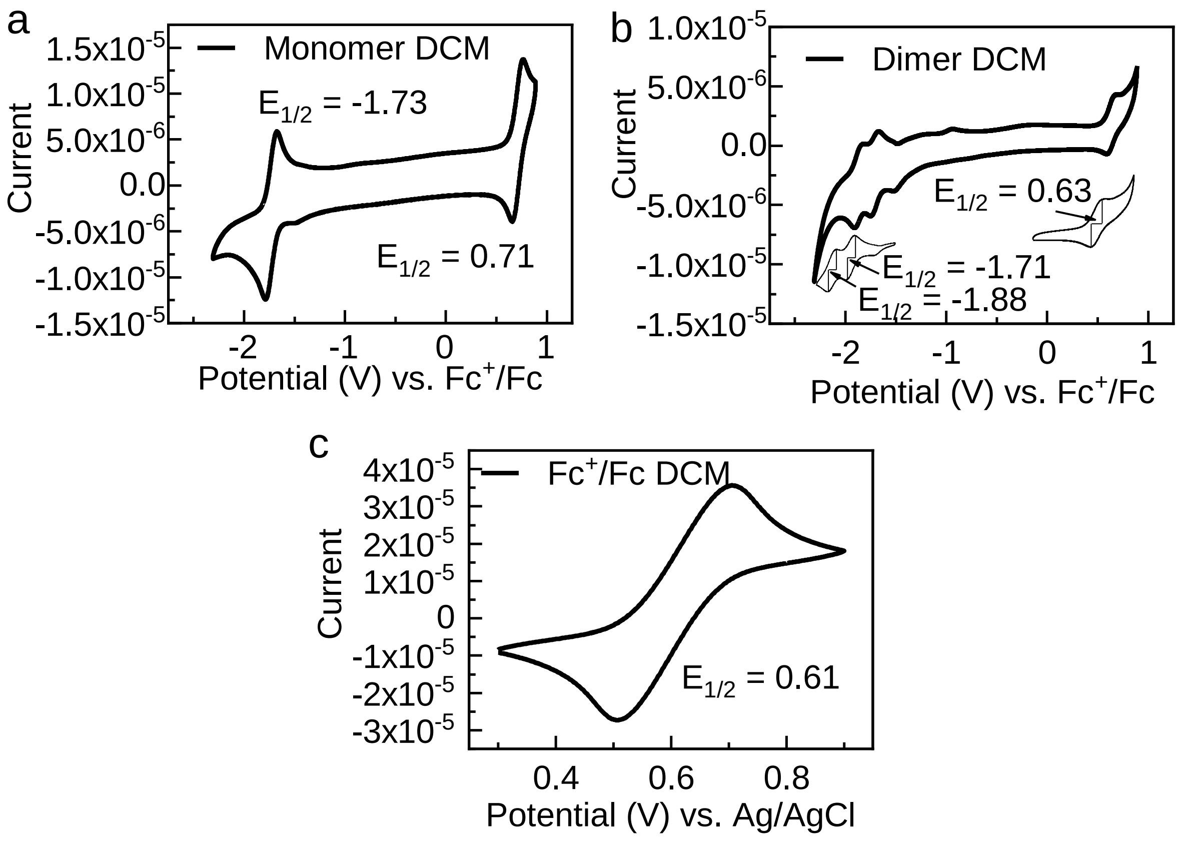

Supplementary Figure 36. Electrochemical analysis of the monomer (a) and the dimer (b) in DCM. Ferrocene (Fc^+/Fc) was measured as an external reference (c).

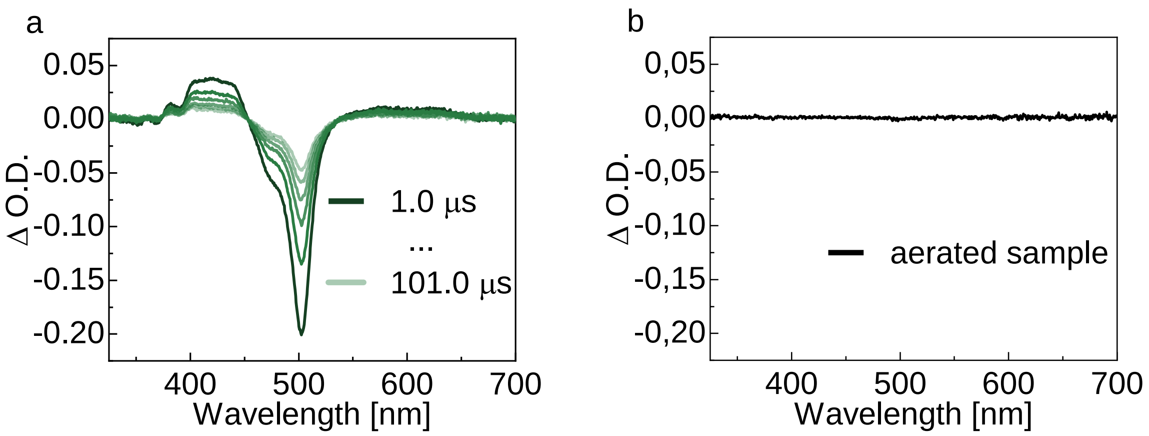

Supplementary Figure 37. Transient absorption measurements of the monomer in ACN (a). Measurements were taken upon excitation at 505 nm, with an integration time of 100 ns. Delay times between 1 µs to 100 µs were used for the measurements. b) No signals could be obtained in the sample after exposure to oxygen.

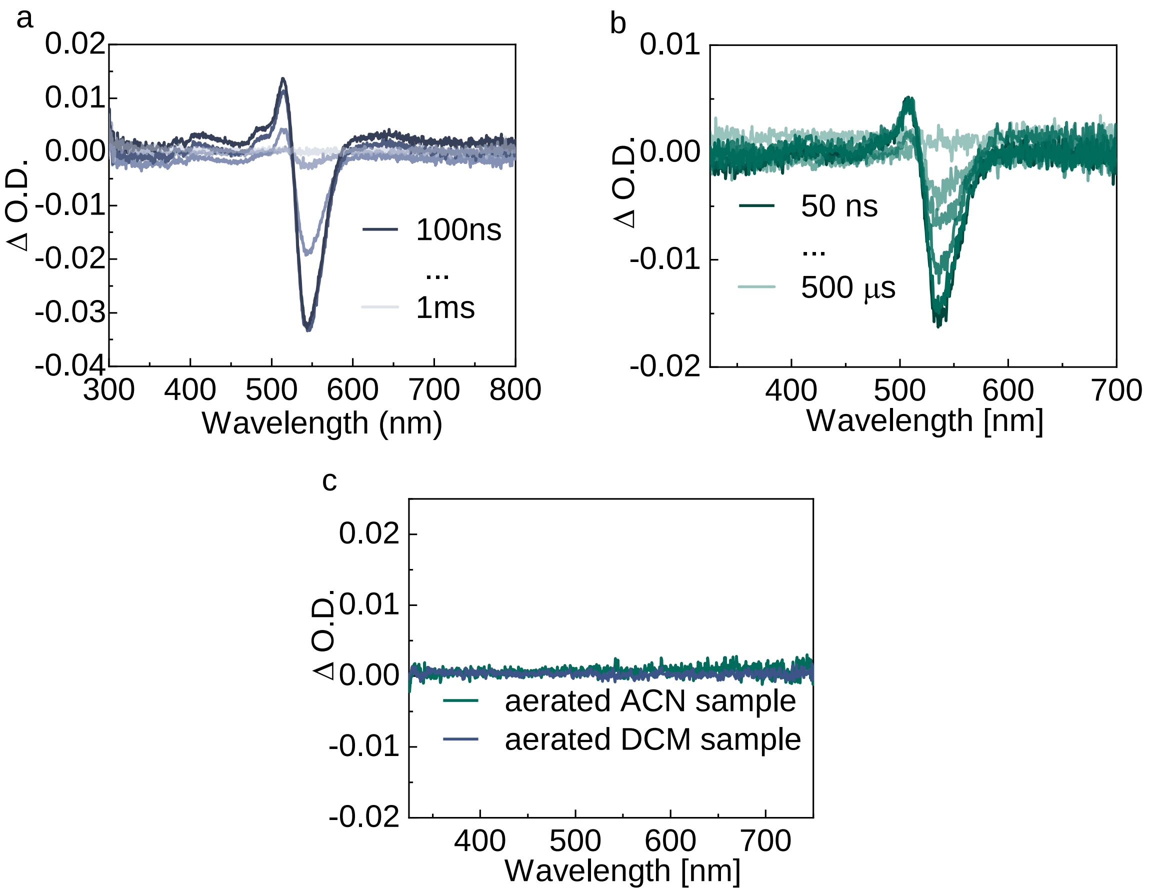

Supplementary Figure 38. Transient absorption measurements of the dimer in DCM (a) and ACN (b). Measurements were taken upon excitation at 542 nm, with an integration time of 100 µs. Delay times between 100 ns to 1 ms were used for the measurements in DCM, and between 50 ns to 500 µs for measurements in ACN. No signals could be obtained in the sample after exposure to oxygen (c).

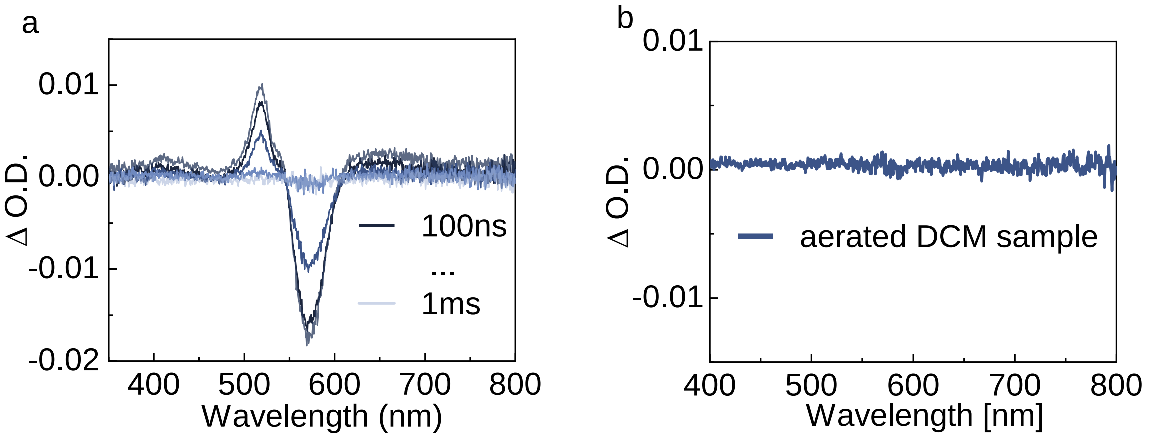

Supplementary Figure 39. Transient absorption measurements of trimer 1 in DCM (a) Measurements were taken upon excitation at 563 nm, with an integration time of 100 µs. No signals could be obtained in the sample after exposure to oxygen (b).

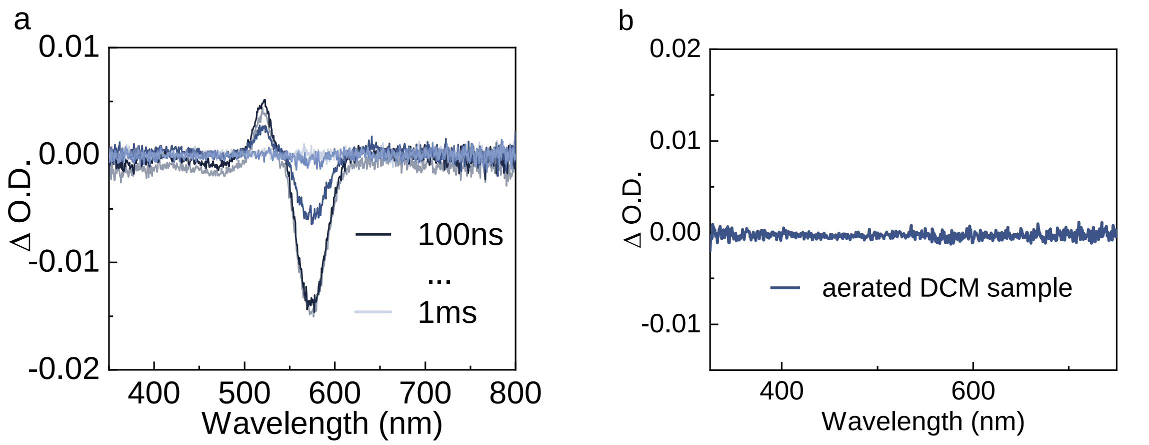

Supplementary Figure 40. Transient absorption measurements of trimer 2 in DCM (a). Measurements were taken upon excitation upon 563 nm, with an integration time of 100 µs. No signals could be obtained in the sample after exposure to oxygen (b).

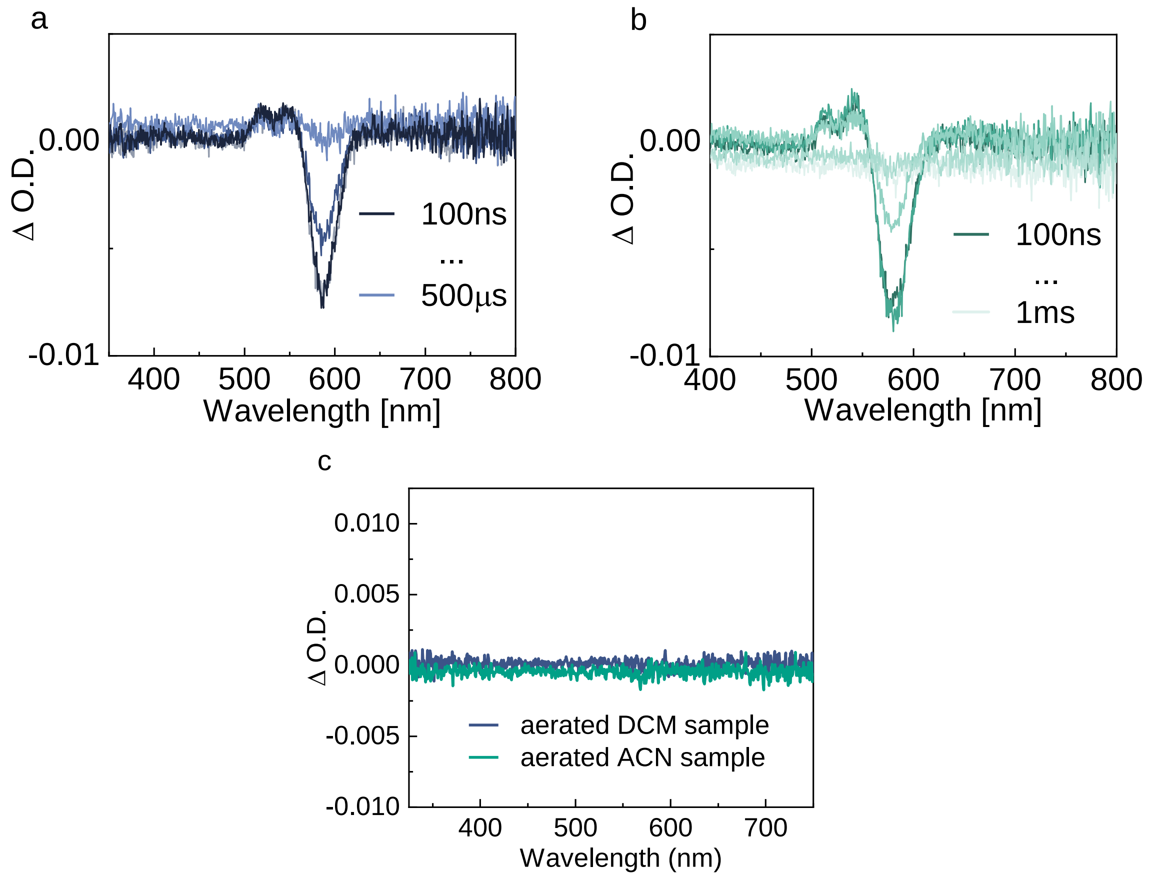

Supplementary Figure 41. Transient absorption measurements of the tetramer in DCM (a) and ACN (b). Measurements were taken upon excitation upon 563 nm, with an integration time of 100 µs. Delay times between 100 ns to 500 µs were used for the measurements in DCM, and between 100 ns to 1 ms for measurements in ACN. No signals could be obtained in the sample after exposure to oxygen (c).

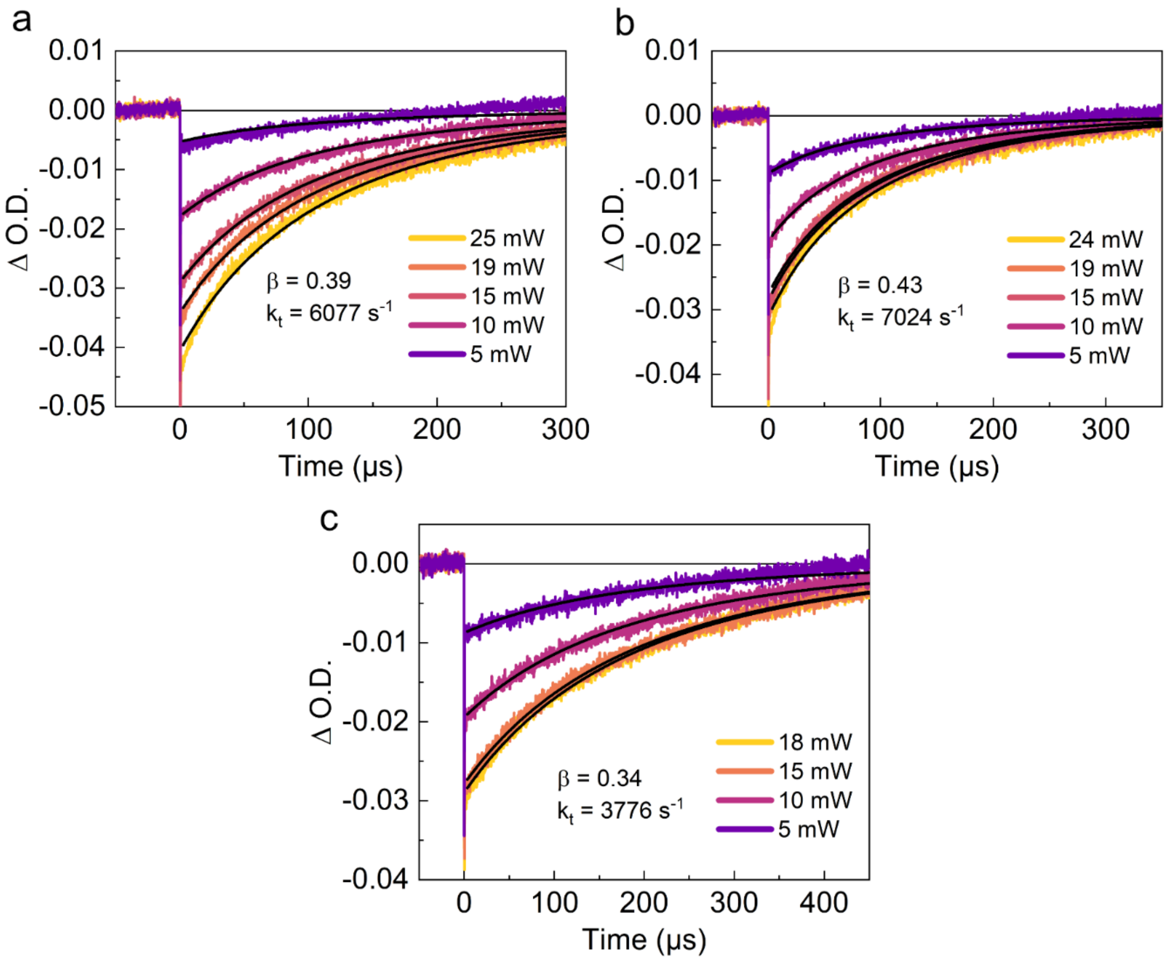

Supplementary Figure 42. Intensity dependent transient absorption measurements of the monomer in a) DCM b) ACN c) Benzonitrile globally fitted using equation 2.

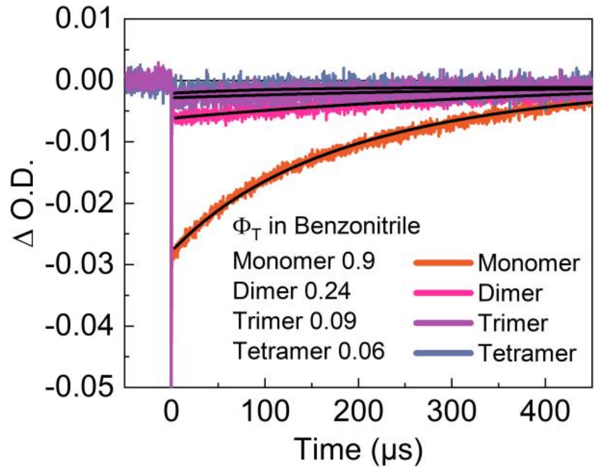

Supplementary Figure 43. Ground state bleach kinetics of the monomer, dimer, trimer and tetramer probed at the respective ground state bleach maxima in benzonitrile.

Femtosecond transient absorption

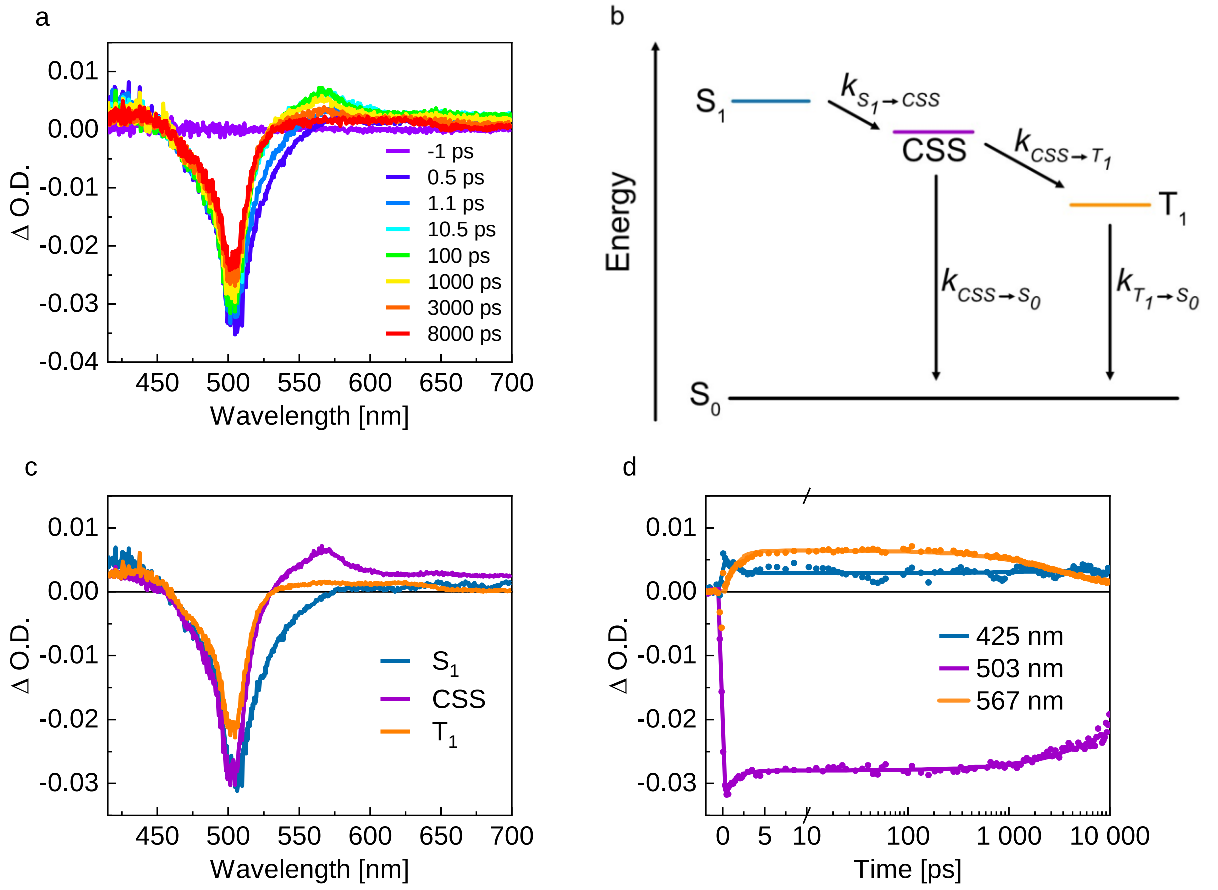

Supplementary Figure 44. fsTA spectra of the monomer in ACN; excitation at 390 nm (a). Kinetic model used to analyse the fsTA data (b). Species-associated spectra of the SVD analysis using the model shown in panel b (c). Selected kinetics at 425 nm, 503 nm, and 567 nm with the model data shown as a solid line (d).

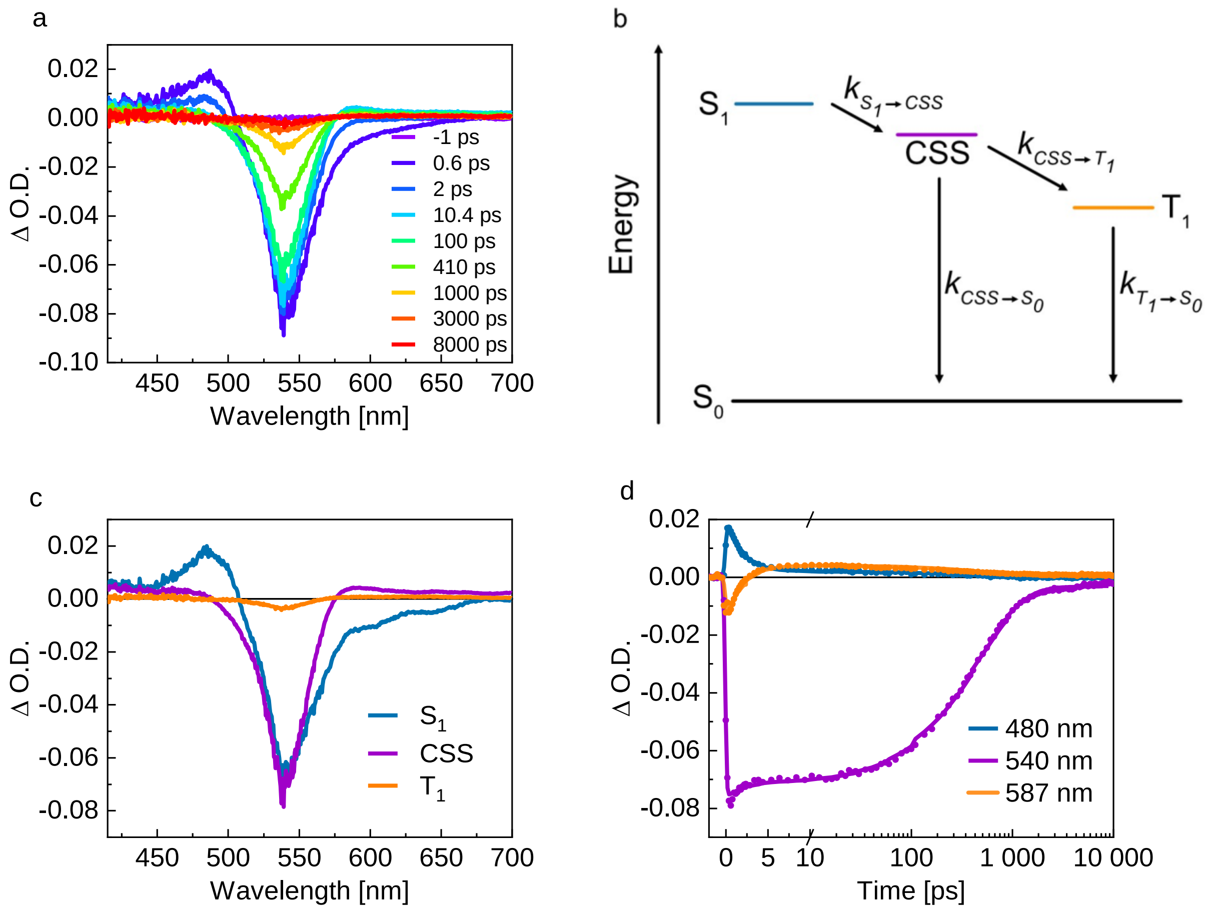

Supplementary Figure 45. fsTA spectra of the dimer in ACN; excitation at 390 nm (a). Kinetic model used to analyse the fsTA data (b). Species-associated spectra of the SVD analysis using the model shown in panel b (c). Selected kinetics at 480 nm, 540 nm, and 587 nm with the model data shown as a solid line (d).

Analyzing recombination using Marcus theory

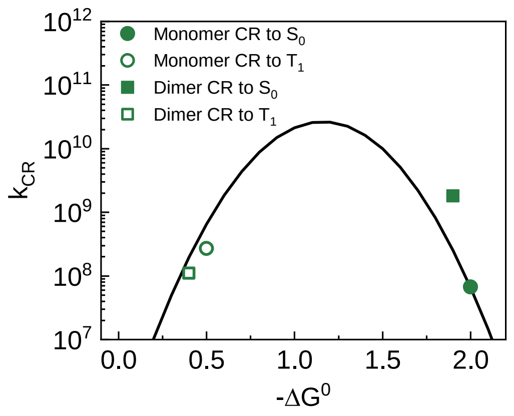

Supplementary Figure 46. Charge recombination rate constants (k_{CR}) from the CS state to the ground and triplet states, showing the dependence on the corresponding free energy change, ΔG^0. Values are taken from Supplementary Table 8.

Supplementary Tables

Supplementary Table 1. The details of the pseudo 2D diffusion experiments. Relax. d. denotes relaxation delay and it is the sum of the acquisition time and the parameter D1 also named as relaxation delay. In the experiment is δ basically the pulse width, which is separated by a time interval (Δ). G.S. stands for gradient strength and G/cm for Gauss/cm.

Sample	Field (MHz)	Δ (ms) max.	δ (ms)	min. G.S. (G/cm)	G.S. (G/cm)	Relax. d. (s)
Monomer	700	50	1.6	1.16	56.98	4.9
Dimer	700	50	1.8	1.16	56.98	4.9
Trimer1	600	50	2.6	1.00	49.45	3.5
Trimer2	600	50	2.6	1.00	49.45	3.5
tetramer	600	50	2.8	1.00	49.45	3.5

Supplementary Table 2. The experimental and simulated diffusion coefficients for the different samples in m^2/s. The column HYDRO++10[16, 17] and HYDROSUB[18] means simulated diffusion coefficients with software named the same. The viscosity for DCM used in the simulation was 0.00413 Poise.[19]

Sample	Experiment	HYDRO++10	HYDROSUB
Monomer	$1.2\ 10^{-9} \pm 3.1 \cdot 10^{-12}$	$1.3\ 10^{-9}$	$1.2\ 10^{-9}$
Dimer	$9.3\ 10^{-10} \pm 9.3 \cdot 10^{-12}$	$8.9\ 10^{-10}$	$8.0\ 10^{-10}$
Trimer1	$6.1\ 10^{-10} \pm 9.8 \cdot 10^{-12}$	$7.1\ 10^{-10}$	$6.2\ 10^{-10}$
Trimer2	$6.2\ 10^{-10} \pm 2.4 \cdot 10^{-11}$	$7.1\ 10^{-10}$	$6.2\ 10^{-10}$
Tetramer	$5.1\ 10^{-10} \pm 6.7 \cdot 10^{-12}$	$6.1\ 10^{-10}$	$5.1\ 10^{-10}$

Supplementary Table 3. Quantum mechanical calculations. The geometry of singlet and triplet ground states were evaluated in vacuum at the ωB97XD/6-31g(d) level of theory (Supplementary Figure 21) and excitation energies were obtained through TD-DFT using Gaussian 16, revision B.01. Energies are reported in eV in relation to the S_0 state.

Sample	S_1	T_1
Monomer	3.044	1.489
Dimer	2.812	1.483
Trimer	2.669	1.475
Tetramer	2.610	1.475

Supplementary Table 4. Electrochemical redox potentials for the monomer and dimer, measured versus Ferrocene as an external reference.

	BODIPY $^{0/\bullet-}$ (V)	anthracene$^{0/\bullet+}$ (V)
Monomer	- 1.73	+ 0.71
Dimer	- 1.71 / - 1.88	+ 0.63

Supplementary Table 5. Calculated CS state energies of the monomer and dimer.

	E_{CS} in Toluene (eV)	E_{CS} in DCM (eV)	E_{CS} in ACN (eV)
Monomer	2.871	2.194	2.007
Dimer	2.771	2.094	1.907

Supplementary Table 6. Values used to simulate the yield of fluorescence and intersystem crossing. To extract the fluorescence quantum yield of the monomer in DCM was the emission deconvoluted in order to remove the contribution from the emission of the CS state. Rates are given in units of s^{-1}, and rates that contain an additional energetic penalty is marked with red (see Supplementary Table 7 for values of activation barriers). Solubility restrictions prevented the Trimer to be dissolved in ACN in large enough quantities to enable absorption matched conditions.

Compound	Φ_{fluo}	Φ_{ISC}	τ_{Em} (ns)	$k_{S1 \to S0_{rad}}$	$k_{S1 \to S0_{nr}}$	$k_{S1 \to CS}$	$k_{CS \to S1}$	$k_{CS \to T1}$	$k_{CS \to S0}$
Monomer (Tol)	0.80		5.29[c]	$1.52 \cdot 10^{8}$	$3.78 \cdot 10^{7}$				
Monomer (DCM)	0.02	0.9	5.17/4.99	$1.52 \cdot 10^{8}$	$3.78 \cdot 10^{7}$	$3.3 \cdot 10^{10}$	$3.3 \cdot 10^{10}$	$1.80 \cdot 10^{8}$	$2.04 \cdot 10^{7}$
Monomer (ACN)	0.01	0.92	5.90/3.11	$1.52 \cdot 10^{8}$	$3.78 \cdot 10^{7}$	$3.3 \cdot 10^{10}$	$3.3 \cdot 10^{10}$	$2.96 \cdot 10^{8}$	$2.55 \cdot 10^{7}$
Dimer (Tol)	0.70		3.05	$2.30 \cdot 10^{8}$	$9.84 \cdot 10^{7}$				
Dimer (DCM)	0.54	0.17	3.68	$2.30 \cdot 10^{8}$	$9.84 \cdot 10^{7}$	$3.3 \cdot 10^{10}$	$3.3 \cdot 10^{10}$	$1.80 \cdot 10^{8}$	$9.17 \cdot 10^{7}$
Dimer (ACN)	0.01	0.14	0.51	$2.30 \cdot 10^{8}$	$9.84 \cdot 10^{7}$	$3.3 \cdot 10^{10}$	$3.3 \cdot 10^{10}$	$2.96 \cdot 10^{8}$	$1.66 \cdot 10^{9}$
Trimer (Tol)	0.72		2.29	$3.14 \cdot 10^{8}$	$1.22 \cdot 10^{8}$				
Trimer (DCM)	0.62	0.1	2.65	$3.14 \cdot 10^{8}$	$1.22 \cdot 10^{8}$	$3.3 \cdot 10^{10}$	$3.3 \cdot 10^{10}$	$1.80 \cdot 10^{8}$	$1.97 \cdot 10^{8}$
Tetramer (Tol)	0.55		1.90	$2.89 \cdot 10^{8}$	$2.37 \cdot 10^{8}$				
Tetramer (DCM)	0.45	0.07	2.09	$2.89 \cdot 10^{8}$	$2.37 \cdot 10^{8}$	$3.3 \cdot 10^{10}$	$3.3 \cdot 10^{10}$	$1.80 \cdot 10^{8}$	$2.98 \cdot 10^{8}$
Tetramer (ACN)	0.07	0.08	0.66	$2.89 \cdot 10^{8}$	$2.89 \cdot 10^{8}$	$3.3 \cdot 10^{10}$	$3.3 \cdot 10^{10}$	$2.96 \cdot 10^{8}$	$1.22 \cdot 10^{9}$

Supplementary Table 7. Relative values of the energy (all energies are given in eV) of S_1 (taken from absorbance spectra), the energy difference between the CS and S_1 states (assuming that the activation barrier fitted in the temperature dependent measurements describes the energy difference between the two states, thus no additional activation barrier exist, and that the CS energy is the same throughout the series), and the rate that contains an additional energetic penalty ($exp(-\Delta E/RT)$).

Compound	Rel. energy of S_1	Relative energy of CS to S_1	Energetic penalty for
Monomer (DCM)	0	-0.12	$k_{CS \to S1}$
Monomer (ACN)	0	-0.31	$k_{CS \to S1}$
Dimer (DCM)	-0.16	0.038	$k_{S1 \to CS}$
Dimer (ACN)	-0.16	-0.15	$k_{CS \to S1}$
Trimer (DCM)	-0.27	0.15	$k_{S1 \to CS}$
Trimer (ACN)	-0.27	-0.04	$k_{CS \to S1}$
Tetramer (DCM)	-0.32	0.20	$k_{S1 \to CS}$
Tetramer (ACN)	-0.32	0.012	$k_{S1 \to CS}$

Supplementary Table 8. Rate constants from global analysis of the fsTA of the monomer and dimer in Supplementary Figures 42 and 43, respectively.

Sample	$k_{S_1 \to CSS}$ (s^{-1})	$k_{CSS \to T_1}$ (s^{-1})	$k_{CSS \to S_0}$ (s^{-1})	$k_{T_1 \to S_0}$ (s^{-1})
Monomer	7.8×10^{11}	2.7×10^{8}	6.7×10^{7}	$< 2 \times 10^{7}$
Dimer	6.8×10^{11}	1.1×10^{8}	1.8×10^{9}	$< 2 \times 10^{7}$

Supplementary References

1. Peng Y-Z, Guo G-C, Guo S, Kong L-H, Lu T-B, Zhang Z-M. Charge Transfer from Donor to Acceptor in Conjugated Microporous Polymer for Enhanced Photosensitization. *Angew Chem Int Ed* 2021, **60**(40)**:** 22062-22069.
2. Rihn S, Erdem M, De Nicola A, Retailleau P, Ziessel R. Phenyliodine(III) Bis(trifluoroacetate) (PIFA)-Promoted Synthesis of Bodipy Dimers Displaying Unusual Redox Properties. *Org Lett* 2011, **13**(8)**:** 1916-1919.
3. Nepomnyashchii AB, Bröring M, Ahrens J, Bard AJ. Synthesis, Photophysical, Electrochemical, and Electrogenerated Chemiluminescence Studies. Multiple Sequential Electron Transfers in BODIPY Monomers, Dimers, Trimers, and Polymer. *J Am Chem Soc* 2011, **133**(22)**:** 8633-8645.
4. Wu D, Chen A, Johnson CS. An improved diffusion-ordered spectroscopy experiment incorporating bipolar-gradient pulses. *J Magn Reson, Ser A* 1995, **115**(2)**:** 260-264.
5. Lewis J, Maroncelli M. On the (uninteresting) dependence of the absorption and emission transition moments of coumarin 153 on solvent. *Chem Phys Lett* 1998, **282**(2)**:** 197-203.
6. Kasha M, Rawls HR, El-Bayoumi MA. The exciton model in molecular spectroscopy. *Pure Appl Chem* 1965, **11**(3-4)**:** 371-392.
7. Yoon ZS, Yoon M-C, Kim D. Excitonic coupling in covalently linked multiporphyrin systems by matrix diagonalization. *J Photochem Photobiol C* 2005, **6**(4)**:** 249-263.
8. Van der Auweraer M, Grabowski ZR, Rettig W. Molecular structure and the temperature-dependent radiative rates in Twisted Intramolecular Charge-Transfer and exciplex systems. *J Phys Chem* 1991, **95**(5)**:** 2083-2092.
9. Wang Z, Zhao J. Bodipy–Anthracene Dyads as Triplet Photosensitizers: Effect of Chromophore Orientation on Triplet-State Formation Efficiency and Application in Triplet–Triplet Annihilation Upconversion. *Org Lett* 2017, **19**(17)**:** 4492-4495.
10. Müller C, Pascher T, Eriksson A, Chabera P, Uhlig J. KiMoPack: A python Package for Kinetic Modeling of the Chemical Mechanism. *J Phys Chem A* 2022, **126**(25)**:** 4087-4099.
11. Barbara PF, Meyer TJ, Ratner MA. Contemporary Issues in Electron Transfer Research. *J Phys Chem* 1996, **100**(31)**:** 13148-13168.
12. Marcus RA. On the Theory of Oxidation-Reduction Reactions Involving Electron Transfer. I. *The Journal of Chemical Physics* 1956, **24**(5)**:** 966-978.
13. Bolton JR, Archer MD. Basic Electron-Transfer Theory. *Electron Transfer in Inorganic, Organic, and Biological Systems*, vol. 228. American Chemical Society, 1991, pp 7-23.
14. Closs GL, Calcaterra LT, Green NJ, Penfield KW, Miller JR. Distance, stereoelectronic effects, and the Marcus inverted region in intramolecular electron transfer in organic radical anions. *J Phys Chem* 1986, **90**(16)**:** 3673-3683.
15. Marcus RA. Electrostatic Free Energy and Other Properties of States Having Nonequilibrium Polarization. I. *The Journal of Chemical Physics* 1956, **24**(5)**:** 979-989.
16. De La Torre JG, Navarro S, Martinez ML, Diaz F, Cascales JL. HYDRO: a computer program for the prediction of hydrodynamic properties of macromolecules. *Biophys J* 1994, **67**(2)**:** 530-531.
17. García de la Torre J, del Rio Echenique G, Ortega A. Improved calculation of rotational diffusion and intrinsic viscosity of bead models for macromolecules and nanoparticles. *J Phys Chem B* 2007, **111**(5)**:** 955-961.
18. Garcia de la Torre J, Carrasco B. Hydrodynamic properties of rigid macromolecules composed of ellipsoidal and cylindrical subunits. *Biopolymers* 2002, **63**(3)**:** 163-167.
19. Acevedo IL, Katz M. Viscosities and thermodynamics of viscous flow of some binary mixtures at different temperatures. *J Solution Chem* 1990, **19:** 1041-1052.
