# Supplementary material for: Lowering of the singlet-triplet energy gap via intramolecular exciton-exciton coupling
Source: Nat Commun. 2024 Oct 8;15:8705. doi: 10.1038/s41467-024-53122-7 (PMC11461719; doi:10.1038/s41467-024-53122-7)
Supplement: Supplementary file 3 — Description of Additional Supplementary Files [file 41467_2024_53122_MOESM3_ESM.pdf]

Description of Additional Supplementary Files:

Supplementary Dataset 1: The raw data for the NMR spectra (fids) that are presented in Supplementary Figures 2-19.

Supplementary Dataset 2: Output files from DFT calculations containing energies and coordinates of the monomer and oligomers in the ground, singlet excited, and triplet states.
